# Pathogenicity and Competitive Fitness of *Salmonella enterica* Serovar 4,[5],12:i:- Compared to *Salmonella* Typhimurium and *Salmonella* Derby in Swine

**DOI:** 10.3389/fvets.2019.00502

**Published:** 2020-01-30

**Authors:** Samantha A. Naberhaus, Adam C. Krull, Bailey L. Arruda, Paulo Arruda, Orhan Sahin, Kent J. Schwartz, Eric R. Burrough, Drew R. Magstadt, Franco Matias Ferreyra, Igor R. H. Gatto, Henrique Meiroz de Souza Almeida, Chong Wang, Amanda J. Kreuder

**Affiliations:** ^1^Department of Veterinary Diagnostic and Production Animal Medicine, College of Veterinary Medicine, Iowa State University, Ames, IA, United States; ^2^Department of Statistics, College of Liberal Arts and Sciences, Iowa State University, Ames, IA, United States; ^3^Department of Veterinary Microbiology and Preventive Medicine, College of Veterinary Medicine, Iowa State University, Ames, IA, United States

**Keywords:** *Salmonella*, 4,[5],12:i:-, Typhimurium, Derby, monophasic, pathogenesis, swine, porcine

## Abstract

Since 2014, *Salmonella* 4,[5],12:i:- has emerged as the most common serovar of *Salmonella enterica* identified from swine samples submitted to veterinary diagnostic laboratories in the United States. To compare the pathogenicity of *S*. 4,[5],12:i:- in swine to the known pathogenic *Salmonella* Typhimurium and lesser pathogenic *Salmonella* Derby, 72 pigs (20 per *Salmonella* serovar treatment and 12 controls) were inoculated with either *S*. Typhimurium, *S*. 4,[5],12:i:-, *S*. Derby, or sham-inoculated and followed for up to 28 days thereafter via rectal temperature, fecal scoring, and fecal culture. Animals were euthanized on days 2, 4, or 28 to determine the gross and histopathologic signs of disease and tissue colonization. The results clearly demonstrate that for the isolates selected, serovar 4,[5],12:i:- possesses similar ability as serovar Typhimurium to cause clinical disease, colonize the tonsils and ileocecal lymph nodes, and be shed in the feces of infected swine past resolution of clinical disease. To compare the competitive fitness of *S*. 4,[5],12:i:- to *S*. Typhimurium in swine when co-infected, 12 pigs were co-inoculated with equal concentrations of both *S*. Typhimurium and *S*. 4,[5],12:i and followed for up to 10 days thereafter. When co-inoculated, serovar 4,[5],12:i:- was consistently detected in the feces of a higher percentage of pigs and at higher concentrations than serovar Typhimurium, suggesting an increased competitive fitness of 4,[5],12:i:- relative to serovar Typhimurium when inoculated simultaneously into naïve pigs. Whole genome sequencing analysis of the isolates used in these studies revealed similar virulence factor presence in all *S*. 4,[5],12:i:- and *S*. Typhimurium isolates, but not *S*. Derby, providing additional evidence for similar pathogenicity potential between serovars 4,[5],12:i:- and Typhimurium. Altogether, this data strongly supports the hypothesis that *S*. 4,[5],12:i:- is a pathogen of swine and suggests a mechanism through increased competitive fitness for the increasing identification of *Salmonella* 4,[5],12:i:- in swine diagnostic samples over the past several years.

## Introduction

*Salmonella* infections in swine, known as salmonellosis, can cause septicemia, enterocolitis, or subclinical infections (1, 2). The septicemic form is often caused by *Salmonella* Choleraesuis and generally has high mortality, low morbidity, and signs including anorexia, fever, lethargy, and dyspnea (1). The enterocolitic form has historically been associated with *Salmonella* Typhimurium and generally has low mortality, high morbidity, and signs including anorexia, fever, lethargy, and diarrhea (1). A large number of serovars have also been associated with subclinical disease, which does not cause overt signs of disease but may be associated with reduced productivity and average daily gain (3), in addition to increasing the risk of contamination of the final product during harvest thereby presenting a food safety concern.

With the characteristics of infection and outcome determined partly by the infecting serovar, a more thorough understanding of the pathogenesis of disease caused by highly prevalent serovars can aid in understanding the expected course of disease and appropriate control measures. In previous years, *S*. Typhimurium was the most commonly reported serovar in humans and swine (4, 5). More recently, however, *S*. 4,[5],12:i:- has been increasingly identified worldwide in humans, swine, cattle, and poultry (4, 6, 7). In fact, *S*. 4,[5],12:i:- has been documented to be more common than *S*. Typhimurium in the U.S. swine population based on data from the National Veterinary Services Laboratory (NVSL) and the Iowa State University Veterinary Diagnostic Laboratory (ISU-VDL) since 2014 (6–8). Although *S*. 4,[5],12:i:- has been reported more frequently, additional research is needed to evaluate the specifics of its pathogenicity in swine. Based on published research using multiple-locus variable number tandem repeat analysis (MLVA), phage typing, pulsed-field gel electrophoresis (PFGE), polymerase chain reaction (PCR), and whole-genome sequencing (WGS), it is highly likely that *S*. 4,[5],12:i:- is a monophasic variant of *S*. Typhimurium (9–16). Thus, *S*. 4,[5],12:i:- could resemble the disease-causing ability of *S*. Typhimurium. However, considering the required involvement of flagella in the pathogenesis of *Salmonella* overall, *S*. 4,[5],12:i:- may have an impaired ability to infect swine and cause disease (17–19) as only one phase of flagellar antigens is expressed in this serovar.

To date, only two studies have been published on the pathogenesis and severity of disease caused by *S*. 4,[5],12:i:- infections in swine, with contradictory findings being reported in relation to fecal scoring and rectal temperature following infection (20, 21). Neither study assessed the gross or histologic pathology associated with inoculation of *S*. 4,[5],12:i:- in swine, although a recent retrospective study found that identification of *S*. 4,[5],12:i:- from clinical specimens was associated with histopathologic evidence of disease (22). While little is known about the pathobiology of *S*. 4,[5],12:i:-, the literature is much more clear on the fact that *S*. Typhimurium is considered to be pathogenic in swine while other common serovars such as *S*. Derby are not known to cause significant enteric disease despite frequent isolation (20, 22, 23). Given the differences in the presentation of salmonellosis based on the infecting serovar as well as inter-study differences, more work is needed, with larger sample sizes and at various ages to better determine the effect of *S*. 4,[5],12:i:- infection in swine, particularly when compared to other serovars with known levels of pathogenicity.

Yet another risk associated with *Salmonella* infections of swine is the potential for persistence of the organism, with subsequent transmission to other pigs or contamination of their environment through shedding in feces (24–26). With *Salmonella* transmission occurring primarily through the fecal-oral route, an improved awareness of the expected shedding pattern would also facilitate appropriate interpretation of fecal culture results and understanding of the course of infection. Many serovars have been documented to cause persistent infections in swine as evidenced by prolonged fecal shedding, including Typhimurium, Derby, Yoruba, and Cubana (27, 28), although the duration of persistence varies with serovar, infecting dose, and host-specific factors (2, 28). In pigs naturally infected with various serovars of *Salmonella*, fecal shedding was highly variable on an individual pig basis in terms of both the pattern and amount of shedding (2). Only one study has been completed to evaluate the persistence of *S*. 4,[5],12:i:- in swine following experimental infection, with detection in the feces of 4/4 swine observed up to 49 days after infection with *S*. 4,[5],12:i:- (20). This data indicates the potential for *S*. 4,[5],12:i:- to cause persistent infections in swine, which may be mediated through colonization of sites other than the large intestine.

The ability to colonize tissues throughout the pig has also been shown to differ by serovar. While *S*. Typhimurium is able to colonize the ileum, ileocecal lymph nodes, tonsils, and mandibular lymph nodes, other serovars such as *S*. Rissen and *S*. Anatum have been shown to colonize a smaller subset of those tissues (29). However, *S*. Typhimurium is less able to colonize non-enteric viscera (liver, spleen, lung) and skeletal muscles when compared to ileum, colon, tonsils, and mesenteric lymph nodes (30). Following experimental infections with *S*. 4,[5],12:i:-, the tonsils, ileocecal lymph nodes, cecal mucosa, and Peyer's patches were all colonized with *Salmonella* on DPI 7 in one study (21) while the tonsils, mesenteric lymph nodes, and intestinal tissues were observed to be colonized with *Salmonella* on DPI 21 and 49 in another study (20). Due to the strong correlation between colonization of various tissues of swine at the time of slaughter and increased risk of contamination of the carcass (29), it is necessary to understand the colonization potential of each serovar and the role of host factors to fully assess the potential public health risk from *Salmonella* infections.

The rise in prevalence of *Salmonella* 4,[5],12:i:- in livestock and humans also raises the question of why the serovar has recently emerged and become increasingly prevalent. *Salmonella* 4,[5],12:i:- has repeatedly been reported to be more highly resistant to antimicrobials relative to *S*. Typhimurium (6, 31, 32), which may provide a significant advantage to its survival in swine operations with extensive antimicrobial drug use. Likewise, *S*. 4,[5],12:i:- is commonly resistant to heavy metals such as zinc and copper, which can be added to livestock feeds as an alternative to antibiotics to reduce bacterial infections (16, 33). With the antimicrobial properties of some heavy metals, it is evident that resistance to them may provide a selective advantage over more susceptible serovars. Another potential mechanism leading to the emergence of the *S*. 4,[5],12:i:- serovar is the ability to outcompete other serovars *in vivo. Salmonella* 4,[5],12:i:-, despite lacking one phase of flagellar antigens, has retained its ability to adhere to and invade porcine intestinal epithelial cells *in vitro* (34). Additionally, a study of 133 monophasic isolates showed that the majority possessed the ability to form biofilms (35); this could enhance the *in vivo* survivability while reducing the effects of antimicrobials on the bacteria (35, 36)*. Salmonella* 4,[5],12:i:- also commonly possesses multiple virulence genes that may contribute to its survival within the host and environment; these genes include but are not limited to *sipC* which is involved in cell adhesion and invasion, *sopB* which promotes the influx of inflammatory cells and fluid secretion involved in diarrhea, and *hilA* which activates the invasion process (37). The combination of biofilm formation, presence of virulence genes involved in pathogenesis, resistance to antimicrobials, and resistance to heavy metals may all function together to provide a selective and competitive advantage to *S*. 4,[5],12:i:- in swine production.

Based on the limited data available, we hypothesized that *Salmonella* 4,[5],12:i:- possesses abilities similar to that of *S*. Typhimurium and greater than that of *S*. Derby in regards to pathogenicity, colonization, and persistence in swine. We also hypothesized that *S*. 4,[5],12:i:- displays a competitive advantage in colonization over *S*. Typhimurium to allow the monophasic serovar to predominate in swine. To address some of the gaps in knowledge related to these hypotheses, three separate animal studies were performed with the following objectives: (1) determine the comparative pathology of *S*. Typhimurium, *S*. 4,[5],12:i:-, and *S*. Derby relative to uninfected pigs; (2) compare the ability of the three serovars to colonize tissues throughout the pig; (3) evaluate the persistence of the three serovars in swine over time; and (4) assess the ability of *S*. 4,[5],12:i:- to outcompete *S*. Typhimurium in co-inoculated pigs. In addition, whole genome sequencing was performed to further characterize the isolates chosen for this work.

## Materials and Methods

### *Salmonella* Isolate Selection

*Salmonella enterica* serovars Typhimurium, 4,[5],12:i:-, and Derby isolates were selected from the collection of clinical isolates submitted to the ISU-VDL. These isolates were originally cultured from clinical samples submitted to the ISU-VDL using standard laboratory protocols (8). Serotyping was completed by the NVSL. Selection of isolates for all studies was based on the following criteria: (1) isolation from clinical samples submitted to the ISU-VDL, (2) originated from 3 to 13 week-old pigs, and (3) association with histopathologic lesions suggestive of salmonellosis. For the pathogenesis study (animal study #2), an isolate of *S*. Typhimurium that had previously been used in a successful animal study (ISU-VDL, unpublished data) that met all of the criteria above was selected. To ensure identification of an appropriate clinical isolate of *Salmonella* 4,[5],12:i:- that had retained its virulence following laboratory passage, a small scale preliminary study was performed using three separate isolates of 4,[5],12:i:- meeting the above criteria (animal study #1). For the competitive fitness animal study (animal study #3), clinical isolates of *S*. Typhimurium and *S*. 4,[5],12:i:- that met the above criteria and exhibited complimentary resistance profiles (S. 4,[5],12:i:- isolate susceptible to ceftiofur (MIC < 1) but resistant to gentamicin (MIC > 16); *S*. Typhimurium isolate susceptible to gentamicin (MIC <1) but resistant to ceftiofur (MIC >8) were utilized for this study. Antimicrobial susceptibility testing was completed using the Sensititre™ system and Sensititre™ Bovine/Porcine MIC Plate (ThermoFisher Scientific, Catalog #BOPO6F) according to Clinical and Laboratory Standards Institute (CLSI) guidelines in the VET08 document (38). Ceftiofur has a veterinary-specific swine breakpoint for respiratory disease pathogens only (*Streptococcus suis, Actinobacillus pleuropneumoniae*, and *Pasteurella multocida*) from which the breakpoint for *Enterobacteriaceae* is currently extrapolated. Gentamicin has a human *Enterobacteriaceae* breakpoint which was utilized to guide breakpoint determinations in swine isolates in this study. Based on the human and veterinary breakpoints available for these antimicrobials, extrapolated breakpoints were used to determine susceptibility, intermediate susceptibility, and resistance for the antibiotics of interest on the Sensititre™ Bovine/Porcine MIC Plate.

### General Culture Conditions, Inoculum Preparation, and Sample Culture

*Salmonella* isolates from clinical cases submitted to the ISU-VDL were stored in brain heart infusion (BHI) broth with 20% glycerin at −80°C. Isolates were removed from the freezer, sub-cultured onto tryptic soy agar with 5% sheep blood (BA) (Remel Products, Lenexa, KS), and incubated for 18–24 h at 35°C. Plates were evaluated for purity of culture prior to use.

#### Inoculum Preparation for *in vivo* Studies

Once pure cultures were obtained, colonies of *Salmonella* were added to Mueller Hinton (MH) broth (BD Diagnostics, Sparks, MD). Based on quantitative *Salmonella* culture completed previously to determine the correlation between the optical density and CFUs of *Salmonella*, a target optical density (OD_600_) of 0.09 would approximately correlate to 1 ×10^8^ CFU/mL; this was the goal of the inoculum. Once the inoculum was prepared, serial dilutions of the inoculum were plated to BA to determine the actual concentration of *Salmonella*, and the remaining inoculum was stored at 4°C for <2 h until administered to the piglets.

#### *Salmonella* Detection and Quantification

Fecal and tissue samples were collected during the animal studies for culture using both quantitative and enrichment techniques. All samples collected for culture, including feces throughout the trial and tissues from necropsy, were stored at 4°C immediately following collection and were then transferred to the freezer (−80°C) within 1–5 h of collection (variation based on day of sampling) until further processing could be completed.

Samples collected for quantification of *Salmonella* were thawed at 37°C until they reached room temperature, weighed, and added to Phosphate Buffered Saline (PBS) (Fisher Scientific, Rochester, MN) to create a 1:10 dilution of each sample. Tissue samples were further ground with a mortar-and-pestle type grinder to facilitate pipetting of samples. Serial dilutions were plated to Xylose-Tergitol-Lysine-4 (XLT4) agar (Remel Products) and then incubated at 35°C without CO_2_. Colonies with morphology characteristic of *Salmonella* were counted daily for 3 days of incubation. Interpretation of standard plate count results used the following criteria: (1) quantification at day 3 was used to calculate concentration in the original sample unless plates were overgrown with normal flora at day 3 in which case counts from previous days were used to calculate concentrations; (2) plates with 25-250 colonies were considered reliably countable and (3) counts were averaged if more than one plate was in the countable range. Samples that had *Salmonella* detected by quantitative culture but below the countable level of 25–250 colonies were listed as 1,000 CFU/mL. Samples that had *Salmonella* detected by enrichment culture but not from quantitative culture were listed as 100 CFU/mL. A minimum of one characteristic and representative colony per sample per pig was confirmed as *Salmonella* using Matrix Assisted Laser Desorption Ionization-Time of Flight-Mass Spectrometry (MALDI-TOF-MS) following manufacturer's recommendations (Bruker Daltonic Inc., Billerica, MA). Per ISU-VDL protocol, a minimum MALDI-TOF-MS confidence score of 2.10 was required for a confirmatory genus level identification.

In addition to quantification by serial dilutions, 0.25 mL of 1:10 dilution of each sample was enriched in 5 mL of Buffered Peptone Water (BPW) (Remel Products). The BPW was incubated for 18–24 h at 35°C without CO_2_ prior to subculture to Brilliant Green agar with Novobiocin (BGN) (BD Diagnostics; Sigma-Aldrich, St. Louis, MO) and XLT4 agar. The BGN and XLT4 agars were incubated at 35°C without CO_2_ for 48 h and were observed for colonies with morphology characteristic of *Salmonella*. A minimum of one colony from enrichment subculture per sample was confirmed as *Salmonella* using MALDI-TOF-MS.

For isolation and identification of each serovar of *Salmonella* following co-inoculation of both Typhimurium and 4,[5],12:i:- from samples in animal study #3, *Salmonella* quantification was performed via standard plate counts on XLT4 supplemented with either ceftiofur or gentamicin [3 types of XLT4 agar: (1) XLT4 agar with gentamicin sulfate (VetOne, Boise, ID) at a concentration of 8 μg/mL to inhibit growth of *S*. Typhimurium, (2) XLT4 agar with ceftiofur in the form of Naxcel® (Zoetis, Parsippany, NJ) at a concentration of 0.5 μg/mL to inhibit growth of *S*. 4,[5],12:i:-, and (3) XLT4 without additional antibiotics]. To ensure the plates were inhibitive to the expected serovar, 1–2 representative colonies per animal per time point were confirmed as *Salmonella* by MALDI-TOF-MS and then were identified at the serovar level using real-time multiplex PCR (rtPCR) (8).

### Animal Studies

#### General Information

All studies involving animals were approved by the Iowa State University Institutional Animal Care and Use Committee (IACUC) prior to initiation (11-16-8391-S). All swine used in the studies were 5 weeks of age at the initiation of the study; this age was selected based on evaluation of the most common age of animals positive for 4,[5],12:i:- from diagnostic samples from pigs with diarrhea and histologic lesions consistent with salmonellosis submitted to the ISU-VDL over the past 8 years as well as successful induction of disease with other serovars of *Salmonella* in this age group (24, 39, 40). All animals were pre-screened as negative for porcine reproductive and respiratory syndrome virus (PRRSV) and porcine epidemic diarrhea virus (PEDV) via pooled PCR testing as well as *Salmonella* negative status via individual enrichment fecal culture and/or PCR on enriched cultures prior to initiation of the study. The pigs were acclimated for 72 h following arrival prior to inoculation during which baseline weights, temperatures, and fecal scores were recorded and pre-inoculation fecal swabs were obtained. Pigs were allocated to treatment groups, and each treatment group was housed in a separate biosecure room for the duration of each study. Throughout the acclimation and study periods, all pigs were fed a non-medicated diet of predominantly corn and soybean meal formulated to meet or exceed the NRC guidelines. Pigs were fed *ad libitum* except for the 12 h prior to inoculation during which all pigs were held off feed. All pigs were euthanized using barbiturate overdose.

#### Scoring Systems: Fecal Consistency, Gross Pathology, and Histopathology

Fecal scoring was standardized across all trials on a scale of 1–5 (1 = dry feces, 2 = moist feces, 3 = mild diarrhea, 4 = severe diarrhea, and 5 = watery diarrhea) as previously described (41). This scoring system is depicted in Supplemental Figure 1. Fecal scores of 1 and 2 were both considered to be normal scores, with a score of 2.5 or above indicating the presence of diarrhea. Pigs were diagnosed with clinical disease when the fecal score was 3 or above and/or rectal temperature was outside of the normal range of 101.5–103.5°F (38.6–39.7°C).

Gross necropsy scoring was completed on animals across all trials in a standardized manner. The severity and distribution of gross lesions were observed along the intestinal tract, with the entire intestinal tract length opened and evaluated for the presence of fibrinous exudate. Samples collected at time of necropsy for histopathologic evaluation were placed in 10% neutral buffered formalin and included the following: liver; spleen; ileocecal lymph nodes; proximal, middle, and distal jejunum; ileum; cecum; middle (or visible lesion) and apex of spiral colon; and rectum. Histologic evaluation was performed at the ISU-VDL by a pathologist who was blinded to pig number and treatment group. The histologic evaluation protocol is summarized in Table 1. The ileocecal lymph node, spleen, and liver were evaluated for the presence or absence of neutrophils in 5-400X fields of view. These tissues were scored on a scale of 1–6 (1 = neutrophils absent in all views, 2 = neutrophils present in 1/5 views, 3 = neutrophils present in 2/5 views, 4 = neutrophils present in 3/5 views, 5 = neutrophils present in 4/5 views, 6 = neutrophils present in all views). The mean neutrophil count from 5-400X fields of view was determined for each small and large intestine sample. Any view with more than 100 neutrophils was considered “too numerous to count” and listed as 100 for averaging purposes. For large intestine samples, the mean was obtained of three crypt depths per section measured at 10X and using an eyepiece micrometer. Lastly, an ulceration score was determined for each small and large intestine sample. The ulceration score was equal to the number of crypts over which the most severe foci extended. This score ranged from 0 to 5, with 0 indicating a lack of observed ulceration and 5 indicating an ulcer spanning 5 or more crypts.

**Table 1:** Summary of histopathologic measurements obtained from each of the evaluated tissue sections during all three animal studies.

**Histopathologic evaluation**	
**Tissue location**	**Obtained measurements**
Proximal jejunum Mid jejunum Distal jejunum Ileum	Neutrophil count^*^ Ulceration score^†^
Cecum Apex of spiral colon Mid spiral colon Rectum	Neutrophil count^*^ Crypt depth^‡^ Ulceration score^†^
Ileocecal lymph node Spleen Liver	Neutrophil presence^§^

*FOV, field of view; TNTC, too numerous to count*.

* *Average of 5 neutrophil counts; at 400X FOV, >100 neutrophils in one FOV was considered TNTC and listed as 100*.

† *Number of crypts over which most severe ulceration foci extended, ranging from 0 to 5 (5 = ulcer spanning five or more crypts)*.

‡ *Average of three crypt depths measured at 10X FOV*.

§ *Presence or absence of neutrophils (in any amount) in 5-400X FOV, ranging from 1 to 5 (1 = no neutrophils observed in 5 FOV, 5 = neutrophils observed in all 5 FOV)*.

A scoring system was derived to obtain an overall score of the histopathology data for each section to facilitate comparison between sections of intestine. For a mean neutrophil count of <5, 0 points were assigned. For every increase by 10 neutrophils, one additional point was assigned (i.e., 1 point for 5–10 neutrophils, 2 points for 11–20 neutrophils, 3 points for 21–30 neutrophils, etc.). Ulceration scores translated directly as points to the overall score (i.e., ulceration score of 2 added 2 points to the overall score). For crypt depths, the scoring system was as follows: <700 μm = 0 points, 700–800 μm = 1 point, 800–900 μm = 2 points, 900–1,000 μm = 3 points, >1,000 μm = 4 points). The crypt depth score was shifted to the right by 100 μm for the rectum due to longer crypts in health (i.e., <800 = 0 points, 800–900 = 1 point, etc.). One additional point was added if evidence of submucosal inflammation was noted. Another point was added if crypt abscesses were observed.

#### Animal Study #1: Preliminary Evaluation of Pathogenicity of Salmonella 4,[5],12:i-

To identify an appropriate clinical isolate of *Salmonella* 4,[5],12:i:- that had retained its pathogenic ability following laboratory passage, a small scale pilot study was performed using three separate isolates of 4,[5],12:i:-, isolates ISU-SAL239-15 (A), ISU-SAL240-15 (B), and ISU-SAL241-16 (C), meeting the above criteria. A total of 9 5 week-old pigs were individually identified and allocated to one of three treatment groups with three pigs per isolate. Each group was then orally inoculated with 10 mL of *Salmonella* 4,[5],12:i:- inoculum with one of the three clinical isolates. The actual concentration of the inoculum was 5.3 ×10^7^ CFU/mL, 6.9 ×10^7^ CFU/mL, and 8.6 ×10^7^ CFU/mL for *S*. 4,[5],12:i:- isolates ISU-SAL239-15 (A), ISU-SAL240-15 (B), and ISU-SAL241-16 (C), respectively. The course of clinical disease as indicated by rectal temperature, fecal score, and fecal *Salmonella* quantification was then followed for 7 days. After 7 days, the animals were euthanized for gross and histologic evaluation. Tissue samples collected at necropsy for *Salmonella* quantification included liver, spleen, tonsil, and ileocecal lymph nodes. The results of this study were utilized to select an isolate of *S*. 4,[5],12:i:- to optimize the results of the large-scale animal study presented below.

#### Animal Study #2: Pathogenicity of Salmonella Typhimurium, 4,[5],12:i:-, and Derby in Swine

To determine the ability of *Salmonella* serovar 4,[5],12:i:- to cause disease and establish a carrier state in swine, 72 5 week-old pigs were utilized in a 28 day study examining the effect and duration of infection with 4,[5],12:i:- when compared to the known highly pathogenic serovar Typhimurium and lesser pathogenic serovar Derby. The pigs were individually identified and allocated to the following treatments: (1) 20 pigs received oral inoculation with 4,[5],12:i:- only (isolate ISU-SAL240-15), (2) 20 pigs received oral inoculation with Typhimurium only (isolate ISU-SAL243-14), (3) 20 pigs received oral inoculation with Derby only (isolate ISU-SAL242-16), and (4) 12 pigs sham-inoculated with sterile MH broth served as negative control. The pigs were housed in pens of four, with five total groups per treatment and three for the control group. The groups for each serovar were housed in separate rooms to ensure no cross contamination between treatments would occur.

Following acclimation, the pigs were inoculated with a standardized dose for all serotypes of 10 mL of a target of 1 ×10^8^ CFU/mL *Salmonella* utilizing a combination of 8 mL oral gavage and 2 mL swabbed directly in the back of the mouth ensuring tonsil exposure as occurs during natural infection with *Salmonella*. The actual inoculum concentrations were 1.44 ×10^8^ CFU/mL, 1.53 ×10^8^ CFU/mL, and 1.94 ×10^8^ CFU/mL for *S*. 4,[5],12:i:-, *S*. Derby, and *S*. Typhimurium, respectively. Daily fecal scores were taken on all animals for the first 7 days to monitor progression of clinical disease; as overt clinical disease was expected to decrease after the first week of infection, bi-weekly fecal scores were taken for the remainder of the study. All pigs had rectal temperatures recorded once daily for the first 7 days and bi-weekly thereafter for the remainder of the study. Fecal samples were collected from the rectum of all pigs at 2 DPI, and all pigs still alive at DPI 4, 7, 14, 21, and 28 for quantitative (direct) and enriched *Salmonella* fecal culture to determine the amount of shedding of *Salmonella* into the environment over time following inoculation.

On DPI 2 and 4, five pigs per treatment group and three control pigs were selected for euthanasia for tissue collection based on the severity of clinical signs (i.e., *Salmonella*-infected pigs demonstrating the most severe clinical signs based on a combination of rectal temperature and fecal score). The remaining pigs after DPI 4 (10 per experimental group; 6 in control group) were allowed to complete the study and were euthanized for sample collection at 28 DPI. At the time of euthanasia, evaluation of gross lesions was completed and samples were collected for histopathologic evaluation of the jejunum, ileum, cecum, colon, ileocecal lymph nodes, tonsils, liver, and spleen to evaluate the progression of clinical disease over time. Additional tissue samples, including ileocecal lymph nodes, tonsils, liver, spleen, and colon contents, were collected at the time of necropsy for quantitative *Salmonella* culture to assess the level of *Salmonella* colonization in these tissues at various points over time following inoculation.

#### Animal Study #3: Competitive Fitness of Salmonella Typhimurium and 4,[5],12:i:- in Swine

To determine if *Salmonella* 4,[5],12:i:- has the ability to outcompete other pathogenic serovars of *Salmonella*, such as Typhimurium, *in vivo*, 12 5 week-old pigs were co-inoculated with a 50:50 mixture of the two serovars. The isolates used for the study were *Salmonella* 4,[5],12:i:- ISU-SAL245-16 and *Salmonella* Typhimurium ISU-SAL244-16, which were selected based on complementary antibiotic resistance profiles in addition to the standard criteria used for all isolates. To compare the pathogenicity of these isolates individually, six additional pigs, three for *S*. Typhimurium and three for *S*. 4,[5],12:i:-, were singly inoculated for a controlled comparison. The co-inoculated pigs were housed in pens of four, with three total groups in a single room, while each of the singly inoculated groups were housed in a single pen of three each in separate rooms. Following acclimation, the pigs were inoculated with a standardized dose of 10 mL of 1 ×10^8^ CFU/mL *Salmonella* utilizing combination of 8 mL oral gavage and 2 mL swabbed directly in the back of the mouth for each serovar for a total of 20 mL for co-inoculated and 10 mL for singly inoculated animals. The actual inoculum concentrations were 1.38 ×10^8^ CFU/mL and 1.73 ×10^8^ CFU/mL for *S*. Typhimurium and *S*. 4,[5],12:i:-, respectively. Following inoculation, daily rectal temperatures and fecal scores were taken on all animals for the first 7 days to monitor progression of clinical disease; twice weekly temperatures and fecal scores were taken for the remainder of the study. Fecal samples were collected from all pigs alive on DPI 1–5, 7, and 10 for quantitative culture of both serovars of *Salmonella*. On DPI 4, five random co-inoculated pigs and all six of the singly inoculated pigs were euthanized and tonsils and ileocecal lymph nodes were collected. The remaining five pigs were euthanized on DPI 10 with collection of tonsils and ileocecal lymph nodes for culture and histopathologic evaluation.

The competition index (CI) was calculated using the following formula: (X–Y)/(X + Y), in which X is the number of *Salmonella* 4,[5],12:i:- colonies and Y is the number of *Salmonella* Typhimurium colonies (42). A CI value that is positive indicates that *Salmonella* 4,[5],12:i:- is more fit while a value that is negative indicates that *Salmonella* Typhimurium is more fit. A CI value closer to 1 or −1 indicates dominance of *Salmonella* 4,[5],12:i:- or *Salmonella* Typhimurium, respectively.

### Statistical Analysis

No statistical analysis was completed for animal study #1 as the goals of the study could be accomplished without statistical analysis. For the data sets from animal studies #2 and #3, the population homogeneity was assessed using the Shapiro-Wilk test and the population variance was assessed using the Brown-Forsythe test. Based on the results from these two tests, either parametric or non-parametric tests were chosen and performed using GraphPad Prism. For brevity, the same statistical test was used for each dependent variable when necessary (i.e., temperature, quantitative culture). For quantitative culture of feces from animal study #2, one-way ANOVA followed by Dunnett's test was used to compare the mean amount of *Salmonella* on a log_10_ basis on DPI 0 to the mean amount of *Salmonella* on all other days, within each serovar. For the rectal temperatures from animal study #2, the Kruskal-Wallis test with Dunn's post test was utilized to compare the mean temperature on each DPI to the day of inoculation (DPI 0) within each serovar. Rectal temperatures, fecal scores, and histologic lesion scores were also compared between serovar groups at each DPI using the GLIMMIX procedure of the SAS System to complete the Tukey-Kramer test. For quantitative fecal culture from animal study #3, Poisson distribution was used to compare *S*. Typhimurium to *S*. 4,[5],12:i:- at each DPI. For all tests, a *P* < 0.05 was deemed statistically significant.

### Whole Genome Sequencing

For the five *Salmonella* isolates utilized in animal studies #2 and #3, pure cultures were grown on MH agar as for the inoculum preparation. DNA extraction was performed using the Invitrogen™ by Life Technologies™ ChargeSwitch® gDNA Mini Bacteria Kit for purification of genomic DNA from gram negative bacteria. This was followed by the preparation of the sequencing libraries with the Illumina® Nextera® XT DNA Library Prep kit according to manufacturer's instructions. Pooled samples were sequenced using paired end reads (2 ×150 read length) on the Illumina MiSeq platform. Demultiplexed sequencing data from each of the isolates was then uploaded to Illumina® Basespace Sequence Hub for analysis. The sequencing data was processed using SRST2 platform to generate multi-locus sequence typing (updated from PubMLST on 11/30/2014), antimicrobial resistance and virulence gene presence, as well as plasmid replicon identification (43). Antimicrobial resistance gene determination within the SRST2 platform was completed using ARG-ANNOT V3 (44), and plasmid replicons were identified through SRST2 using PlasmidFinder (45). The virulence factor database utilized by SRST2 was generated from VFDB (http://www.mgc.ac.cn/VFs/) on 11/30/2014 for identification of the presence of known virulence factors in *Salmonella* (46). Draft genome assembly was then completed using SPAdes Genome Assembler (Version 3.9.0) (47).

## Results

### Animal Study #1: *In vivo* Evaluation and Selection of a Clinical Isolate of *S*. 4,[5],12:i:- for Pathogenicity Animal Study

#### Clinical Disease

**Figure 1:**
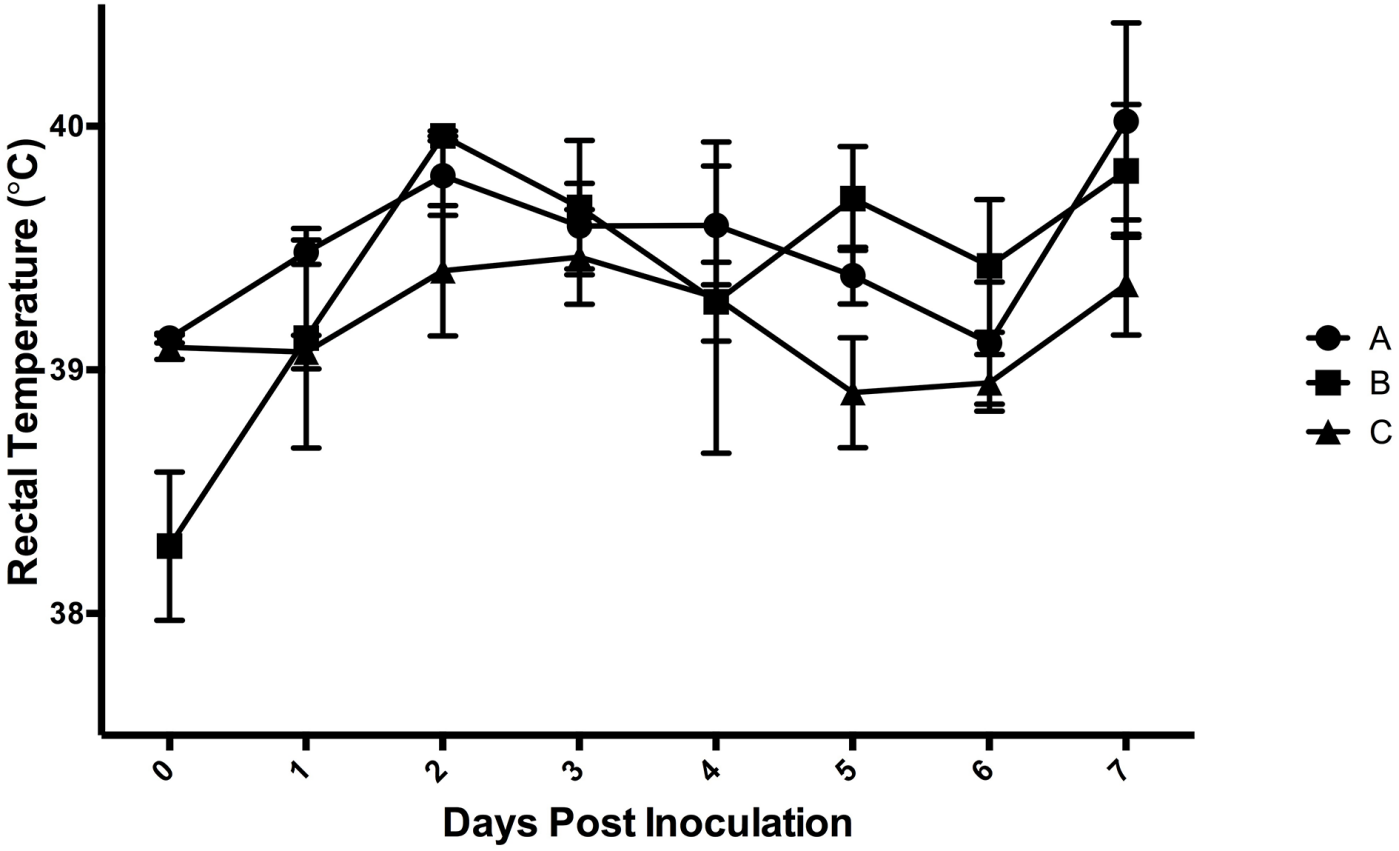
Mean rectal temperatures from animal study #1 following inoculation on DPI 0 with three separate isolates of *S*. 4,[5],12:i:- (A, B, C). The mean and standard error are represented by the vertical bars.

**Figure 2:**
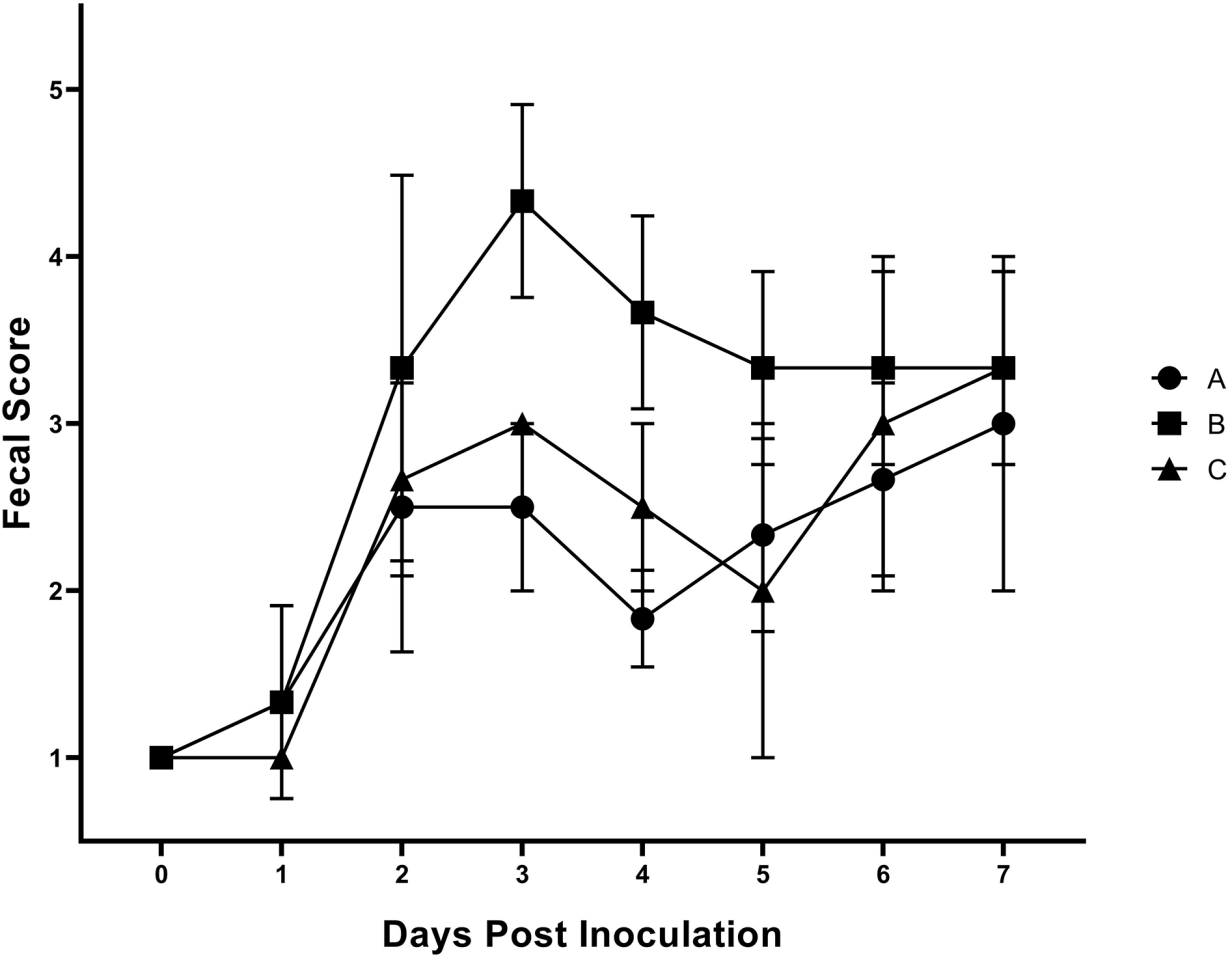
Mean fecal scores from animal study #1 following inoculation on DPI 0 with three separate isolates of *S*. 4,[5],12:i:- (A, B, C). The fecal scoring system ranged from 1 to 5, with 1–2 being considered normal and 5 being considered severe diarrhea. The mean and standard error are represented by the vertical bars.

Results of the preliminary animal study comparing three separate isolates of *Salmonella* 4,[5],12:i:- demonstrated that clinical disease, as indicated by a rectal temperatures outside of the normal range of 101.5–103.5°F (38.6–39.7°C) (Figure 1) and/or diarrhea (fecal score ≥3) (Figure 2), was induced by all isolates, although isolates A and B caused an increase in both rectal temperature and in fecal score, while isolate C caused an increase in the fecal score only. Fecal scores and temperatures from the three isolates, averaged among each group, reached their peak at DPI 3 and 2, respectively. There was detectable shedding of *Salmonella* in the feces with direct culture throughout the entire 7 day study in piglets inoculated with isolates A and B. For the group inoculated with isolate C, mild clinical disease was noted with *Salmonella* shedding only detectable through DPI 4 in pre-enrichment feces. However, *Salmonella* was detectable in enriched fecal samples from all pigs at all time points for all groups inoculated with each of the three isolates (Table 2).

**Table 2:** Culture results of three separate isolates of *Salmonella* 4,[5],12:i:- from animal study #1.

	**Culture positives**^**†**^						
**Inoculum**	**Fecal samples (DPI)**			Necropsy samples^*^			
	**2**	**4**	**7**	**Liver**	**Spleen**	**IC LN**	**Tonsils**
*S*. 4,[5],12:i:- (A)	100%	100%	100%	33%	0%	100%	100%
	3/3	3/3	3/3	1/3	0/3	3/3	3/3
*S*. 4,[5],12:i:- (B)	100%	100%	100%	0%	0%	100%	33%
	3/3	3/3	3/3	0/3	0/3	3/3	1/3
*S*. 4,[5],12:i:- (C)	100%	100%	100%	33%	33%	100%	100%
	3/3	3/3	3/3	1/3	1/3	3/3	3/3

*DPI, days post inoculation; IC LN, ileocecal lymph node*.

* *Necropsy samples were collected on DPI 7*.

† *Positives were positive for Salmonella from quantitative culture and/or enrichment culture*.

#### Bacterial Culture

Ileocecal lymph nodes, tonsils, spleen, and liver were collected at the time of necropsy on DPI 7, with culture results listed in Table 2. The spleen and liver proved to be a poor sample type for the detection of *Salmonella* 4,[5],12:i:- infection as only one out of nine and two out of nine pigs, respectively, were positive for *Salmonella* by quantitative and/or enrichment culture. Ileocecal lymph nodes and tonsils proved to be a better sample for detection of *Salmonella* 4,[5],12:i:- as nine out of nine and seven out of nine pigs, respectively, were positive for *Salmonella* by direct and/or enrichment culture.

#### Gross and Histopathologic Lesions

**Figure 3:**
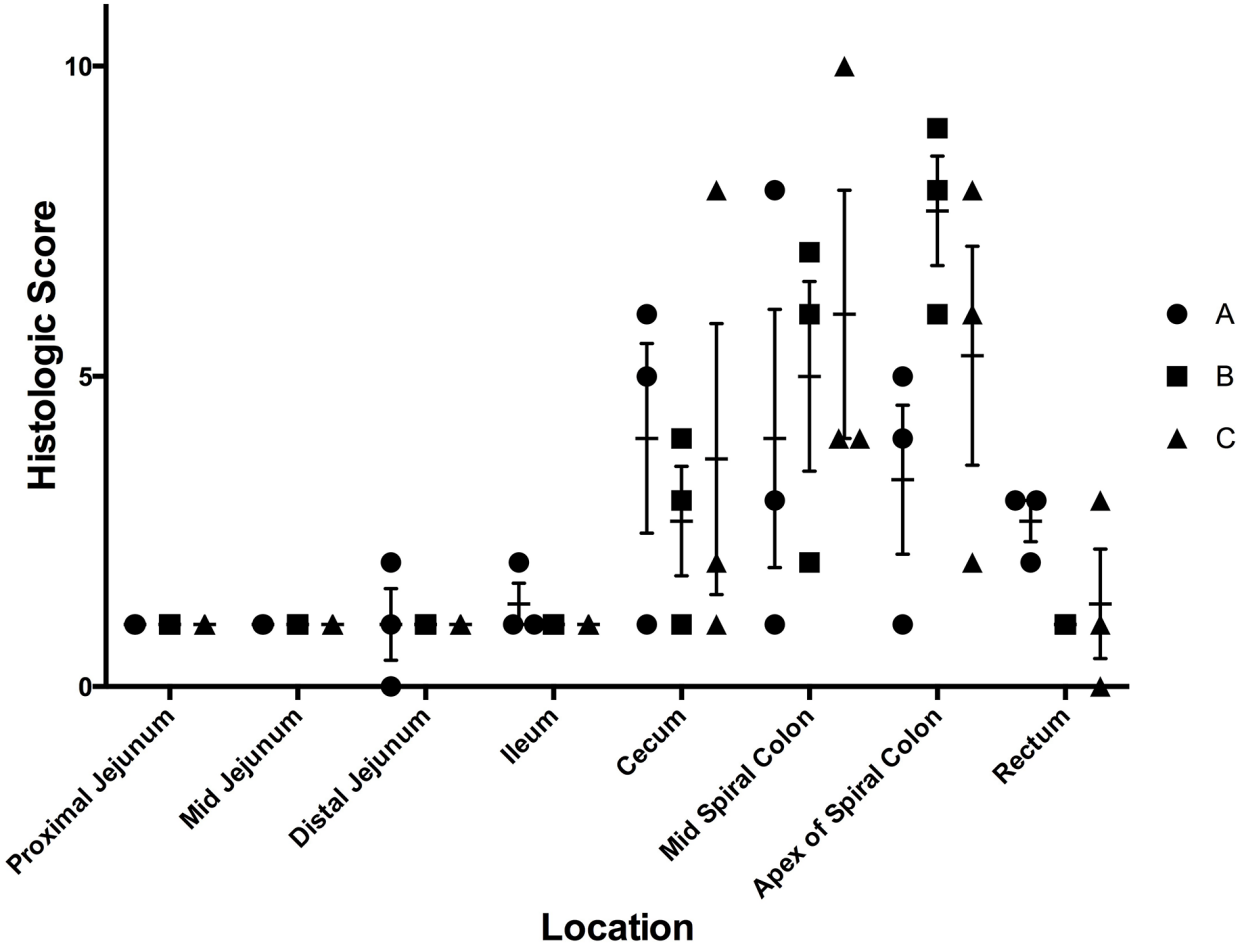
Histologic scores from samples collected at necropsy on day 7 following inoculation with three separate isolates of *S*. 4,[5],12:i:- (A, B, C) in animal study #1. These scores depict the average histologic lesions, as determined by the ulceration, neutrophil infiltration, and crypt elongation and abscessation, and submucosal inflammation, at the time of necropsy on DPI 7. The mean of each isolate-tissue location combination is represented by the horizontal bar with the standard error represented by the vertical line.

Although *Salmonella* 4,[5],12:i:- was detectable in the feces and tissues of the majority of pigs, only one out of nine pigs had gross lesions suggestive of salmonellosis, which consisted of fibrinous colitis; this pig was inoculated with isolate B. Histopathologic evaluation of intestinal tissues on the basis of neutrophil infiltration, crypt elongation, and ulceration revealed that the cecum and spiral colon were the primary target of *Salmonella* 4,[5],12:i:-, regardless of the specific isolate involved in infection, with minimal to no lesions in the small intestine and rectum (Figure 3). Based on these data, isolate B of *S*. 4,[5],12:i:- was selected for the full scale animal study described below.

### Animal Study #2: Comparison of the Pathogenicity of *S*. 4,[5],12:i:-, *S*. Typhimurium, and *S*. Derby in Swine

A total of 70 of the 72 pigs completed the study and were included in the final analysis. One pig in the *S*. Derby group was removed from the study due to premature death on DPI 2. The pig was submitted to the ISU-VDL and determined to have died from a vitamin E-selenium responsive nutritional cardiomyopathy, better known as Mulberry Heart Disease. The other pig removed from the study was among the control group pigs euthanized on DPI 2 and was removed from the study due to the presence of *Isospora suis*, a primary pathogen of swine, detected during histopathologic evaluation. All other animals completed the study and were included in the analysis.

#### Clinical Disease

**Figure 4:**
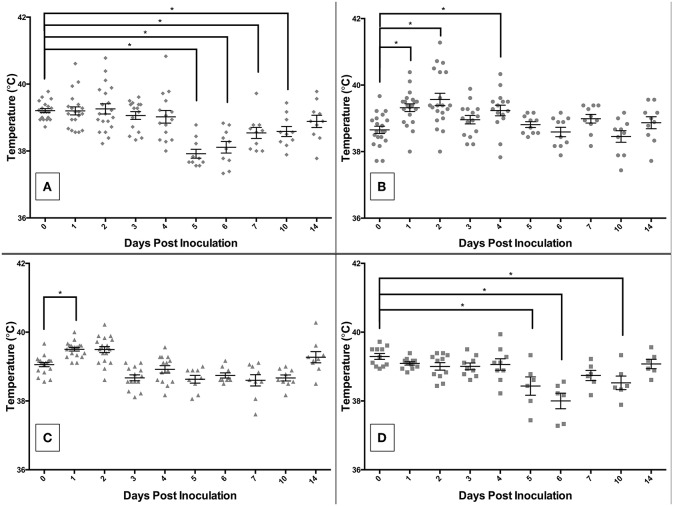
Comparison of rectal temperatures of pigs inoculated with *Salmonella* serovars 4,[5],12:i:- **(A)**, Typhimurium **(B)**, and Derby **(C)**, and non-inoculated control **(D)** pigs in animal study #2. Asterisks represent significant differences from the mean temperature on DPI 0 (*p* < 0.05). Each datum point represents the temperature in individual animal; short horizontal bar represents mean temperature within a group.

The rectal temperature results are shown in Figures 4A–D. Those infected with *S*. 4,[5],12:i:- had a mean temperature significantly different from DPI 0 on DPI 5, 6, 7, 10, and 17 (Figure 4A). The mean temperature (±SE) of pigs infected with *S*. Typhimurium peaked on DPI 2 at 103.2°F (±0.3) (38.5°C ± 0.2), with a statistically significant increase relative to DPI 0 on DPI 1, 2, and 4 (Figure 4B). *Salmonella* Derby-inoculated pigs developed the highest mean temperature on DPI 1 at 103.1°F (±0.1) (39.5°C ± 0.1), with a significant difference from DPI 0 on DPI 1 only (Figure 4C). The control pigs also had a mean rectal temperature significantly different from DPI 0 on DPI 5, 6, and 10 (Figure 4D). Interestingly, there were a number of animals with temperatures that fell outside the normal range, both higher and lower than normal, in the *S*. Typhimurium and *S*. 4,[5],12:i:- groups, but not the control or *S*. Derby groups.

**Figure 5:**
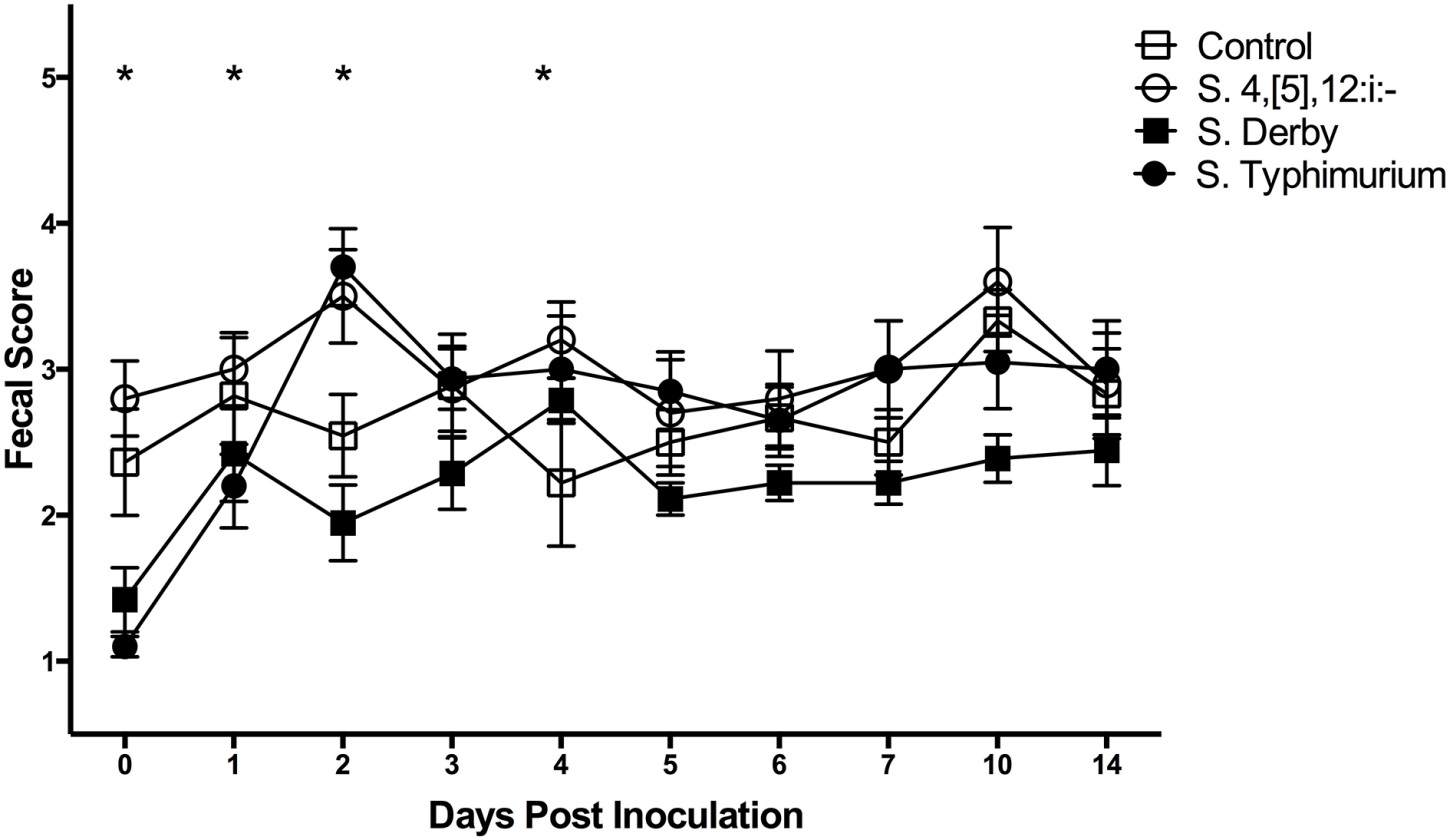
Comparison of fecal scores of pigs inoculated with *Salmonella* serovars 4,[5],12:i:-, Typhimurium, and Derby and non-inoculated control pigs in animal study #2. Symbols represent the mean, with vertical bars representing standard error of the mean. Fecal scores of 1–2 are normal, 3 is mild diarrhea, 4 is moderate diarrhea, and 5 is severe diarrhea. Days with significant differences present are represented by asterisks and are detailed in the text.

The fecal score results are shown in Figure 5. The mean fecal score (±SE) of *S*. Typhimurium-infected pigs peaked on DPI 2 at 3.7 (±0.3) and *S*. 4,[5],12:i:- -infected pigs peaked on DPI 2 at 3.5 (±0.3). The *S*. Derby infected pigs reached a mean fecal score on DPI 4 of 2.8 (±0.2) with all other mean fecal scores being at or below 2.4, therefore indicating that *S*. Derby did not successfully induce prolonged diarrhea in the pigs with the exception of DPI 4. The sham-inoculated control pigs reached a peak fecal score on DPI 28 at 3.5 (±0.2), but also had elevated mean fecal scores on DPI 10 at 3.3 (±0.2) and DPI 3 at 2.9 (±0.4). Several statistically significant differences in the fecal scores between groups were also noted. On DPI 0, *Salmonella* 4,[5],12:i:- had a significantly increased mean fecal score relative to *S*. Derby and *S*. Typhimurium-infected pigs. The mean fecal score of *S*. 4,[5],12:i:- pigs was significantly increased relative to *S*. Typhimurium on DPI 1 and *S*. Derby and the controls on DPI 2. The mean fecal score of *S*. Typhimurium pigs was also significantly increased relative to *S*. Derby and the controls on DPI 2. *Salmonella* 4,[5],12:i:- pigs had a significantly higher mean fecal score relative to the controls on DPI 4.

#### Bacterial Culture

**Figure 6:**
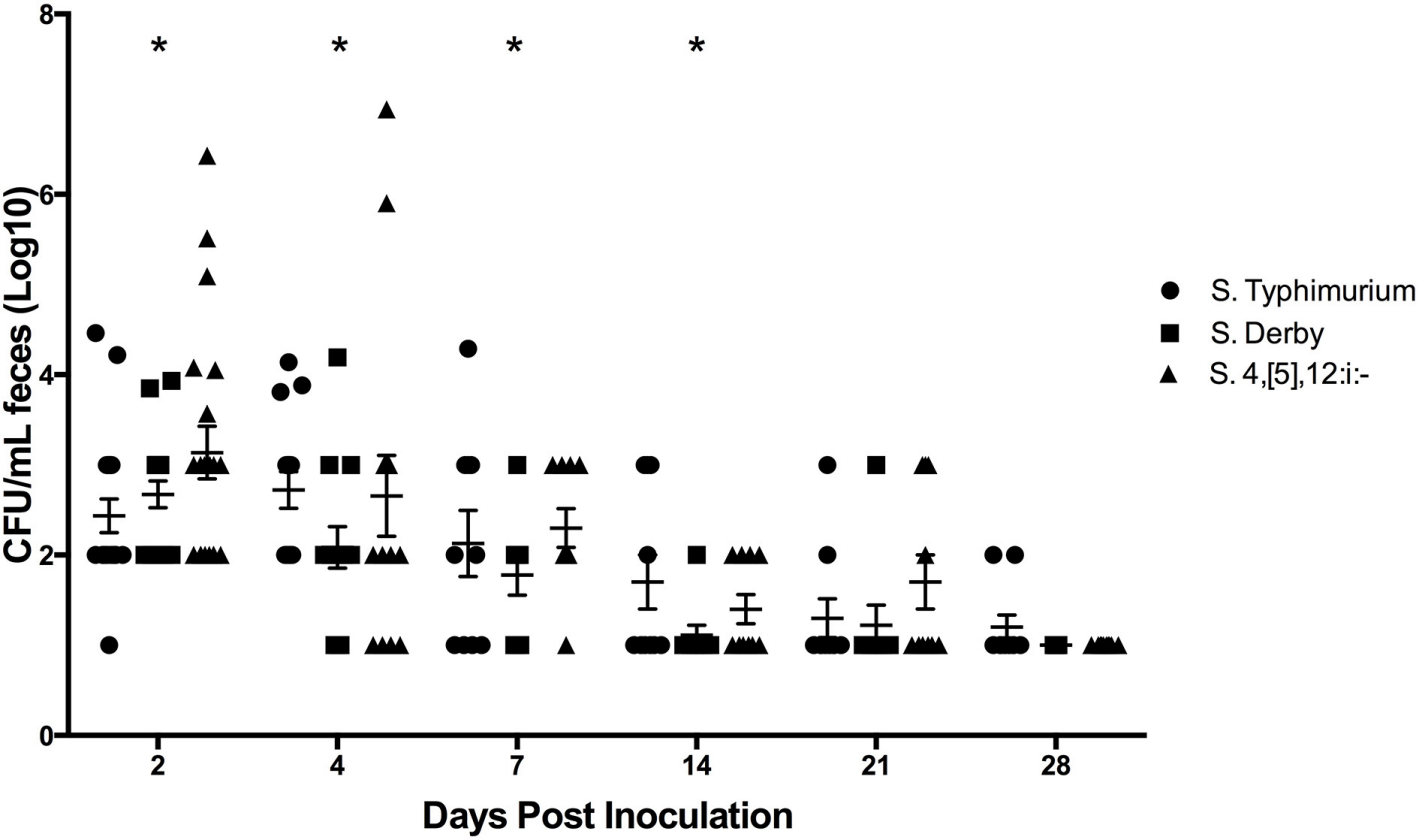
Comparison of quantitative culture results for *Salmonella* in feces collected from pigs inoculated with *Salmonella* serovars 4,[5],12:i:-, Typhimurium, and Derby in animal study #2. The mean CFU/mL and standard error are represented by the horizontal and vertical lines, respectively. Statistically significant differences (*p* < 0.05) were determined within each group compared to DPI 0 at which time no *Salmonella* was detectable. Days with significant differences present from DPI 0 are represented by asterisks and were as follows: *S*. 4,[5],12:i:- on DPI 2, 4, and 7; *S*. Derby on DPI 2, 4, and 7; *S*. Typhimurium on DPI 2, 4, 7, and 14.

All pre-inoculation fecal samples were negative for *Salmonella* via enriched culture and PCR. Throughout the duration of the study, all samples collected from the control pigs were confirmed negative for *Salmonella* by enrichment culture. Fecal culture results are summarized in Supplemental Table 1 and depicted in actual log_10_ CFU/mL in Figure 6. The mean amount of *Salmonella* shed in the feces peaked at 3.0 (±0.1) log_10_ CFU/mL on DPI 4 in *S*. Typhimurium infected pigs. Pigs infected with *S*. 4,[5],12:i:- reached a peak level of *Salmonella* in feces on DPI 2 at 3.4 (±0.2) log_10_ CFU/mL while those infected with *S*. Derby also reached a peak level on DPI 2 at 2.9 (±0.1) log_10_ CFU/mL. All serovar groups had a significantly increased amount of *Salmonella* in the feces on DPI 2, 4, and 7 relative to DPI 0, and *S*. Typhimurium-infected pigs also had a significantly increased amount in the feces on DPI 14. Enriched feces remained positive for *Salmonella* in the *S*. Typhimurium group in 20% of pigs (2 of 10) on DPI 28, while the *S*. 4,[5],12:i:- group only remained positive until DPI 21, at which time 30% of fecal samples (3 of 10) were positive.

Samples collected at necropsy on DPI 2, 4, and 28 were also cultured for the presence of *Salmonella* (Table 3). On DPI 2 and 4, all *Salmonella*-inoculated pigs, with the exception of one *S*. Derby-inoculated pig on DPI 4, had detectable levels of *Salmonella* in their colon contents. The liver and spleen of *Salmonella*-infected pigs had variable results based on infecting serovar and timing after inoculation, ranging from 60% of *S*. Typhimurium-inoculated pigs positive in the liver on DPI 2 while *S*. Derby-inoculated pigs were negative for *Salmonella* in the spleen and liver at all necropsy time points. The ileocecal lymph nodes were positive on DPI 2 and 4 from all *Salmonella*-inoculated pigs. However, the lymph nodes were only positive in 50% (5 of 10) of *S*. 4,[5],12:i:- -inoculated pigs, 67% (6 of 9) of *S*. Derby-inoculated pigs, and 40% (4 of 10) of *S*. Typhimurium-inoculated pigs on DPI 28. Tonsils were positive from all *Salmonella*-infected pigs on DPI 2 and 4, except for three *S*. Derby-inoculated pigs on DPI 4 and one *S*. Typhimurium-inoculated pig on DPI 2. On DPI 28, tonsils were positive for *Salmonella* from 90% (9 of 10) of *S*. 4,[5],12:i:- -inoculated pigs, 67% (6 of 9) of *S*. Derby-inoculated pigs, and 40% (4 of 10) of *S*. Typhimurium-inoculated pigs.

**Table 3:** Comparison of *Salmonella* culture results from samples collected during necropsy from pigs inoculated with *Salmonella* serovars 4,[5],12:i:-, Typhimurium and Derby in animal study #2.

		Culture positives^*^				
**Inoculum**	**Necropsy DPI**	**Location**				
		**Colon**	**Liver**	**Spleen**	**IC LN**	**Tonsils**
S. 4,[5],12:i:-	2	100%	40%	20%	100%	100%
		5/5	2/5	1/5	5/5	5/5
	4	100%	40%	20%	100%	100%
		5/5	2/5	1/5	5/5	5/5
	28	10%	0%	0%	50%	90%
		1/10	0/10	0/10	5/10	9/10
*S*. Derby	2	100%	0%	0%	100%	100%
		5/5	0/5	0/5	5/5	5/5
	4	80%	0%	0%	100%	40%
		4/5	0/5	0/5	5/5	2/5
	28	0%	0%	0%	67%	67%
		0/9	0/9	0/9	6/9	6/9
*S*. Typhimurium	2	100%	60%	20%	100%	80%
		5/5	3/5	1/5	5/5	4/5
	4	100%	20%	0%	100%	100%
		5/5	1/5	0/5	5/5	5/5
	28	30%	0%	0%	40%	40%
		3/10	0/10	0/10	4/10	4/10

DPI, days post inoculation; IC LN, ileocecal lymph nodes.

* *Positives were positive for Salmonella from quantitative and/or enrichment culture*.

#### Gross Lesions

**Figure 7:**
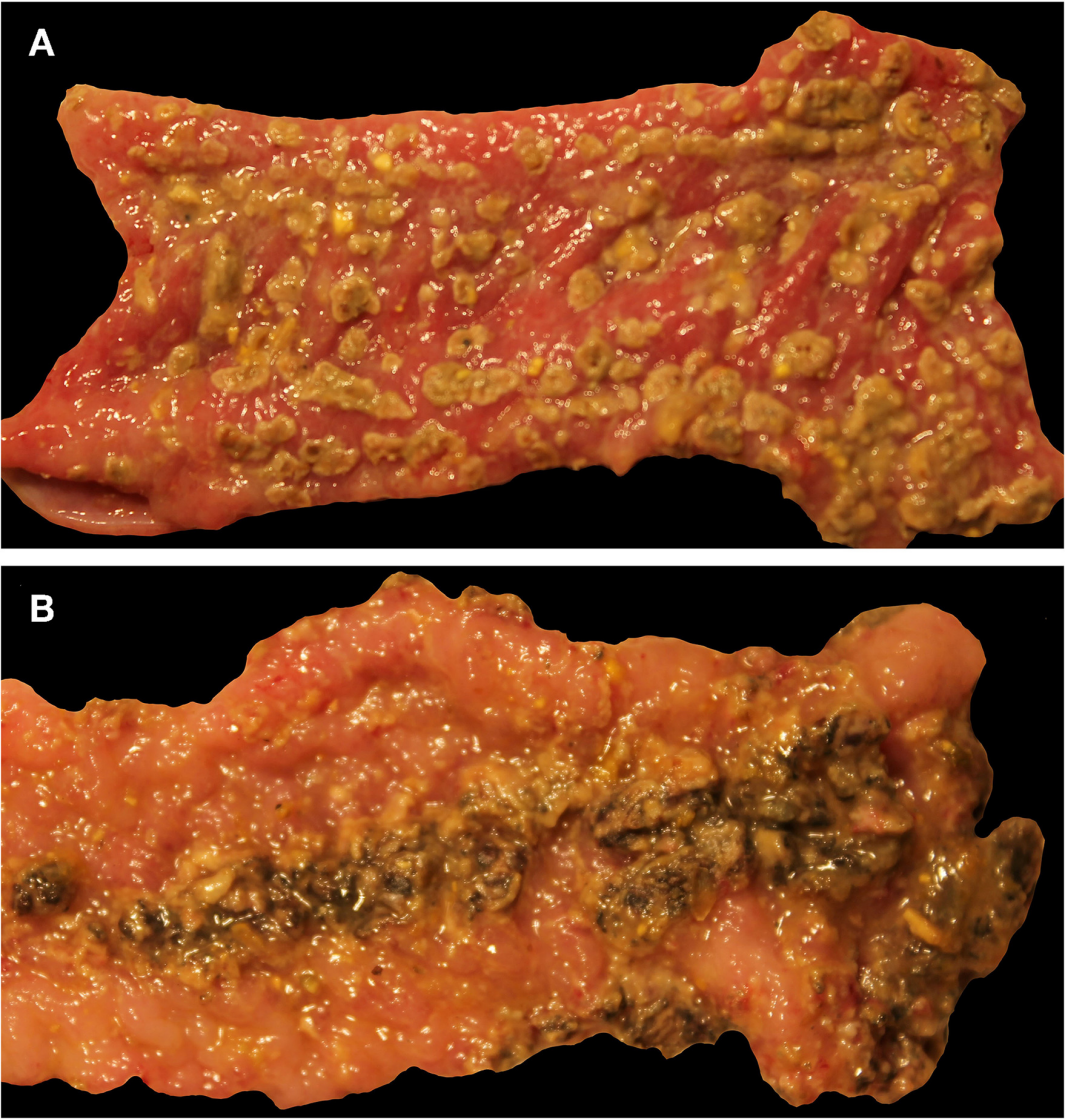
Representative gross lesions following infection with *Salmonella* 4,[5],12:i:- and *Salmonella* Typhimurium. Spiral colon. **(A)** Pig inoculated with *Salmonella* 4,[5],12:i:-, DPI 4, severe diffuse fibrinous colitis. **(B)** Pig inoculated with *Salmonella* Typhimurium, DPI 4, severe diffuse fibrinous colitis.

Gross lesions suggestive of salmonellosis (i.e., fibrinous colitis) were absent in all control pigs and *S*. Derby-infected pigs necropsied on DPI 2 but were present in two out of five pigs inoculated with *S*. Typhimurium and two out of five inoculated with *S*. 4,[5],12:i:-. At DPI 4, similar results were found, with two out of five *S*. 4,[5],12:i:- -inoculated pigs and four out of five *S*. Typhimurium-inoculated pigs possessing gross lesions suggestive of salmonellosis while none of the control or *S*. Derby-inoculated pigs had gross lesions. Representative gross lesions from *S*. 4,[5],12:i:- and *S*. Typhimurium-inoculated pigs on DPI 4 are shown in Figures 7A,B, respectively. However, by DPI 28, only one of 10 *S*. Typhimurium-inoculated pigs and one of 10 *S*. 4,[5],12:i:- -inoculated pigs had gross lesions suggestive of salmonellosis while none of the control or *S*. Derby-inoculated pigs had gross lesions visible.

#### Histopathology

**Figure 8:**
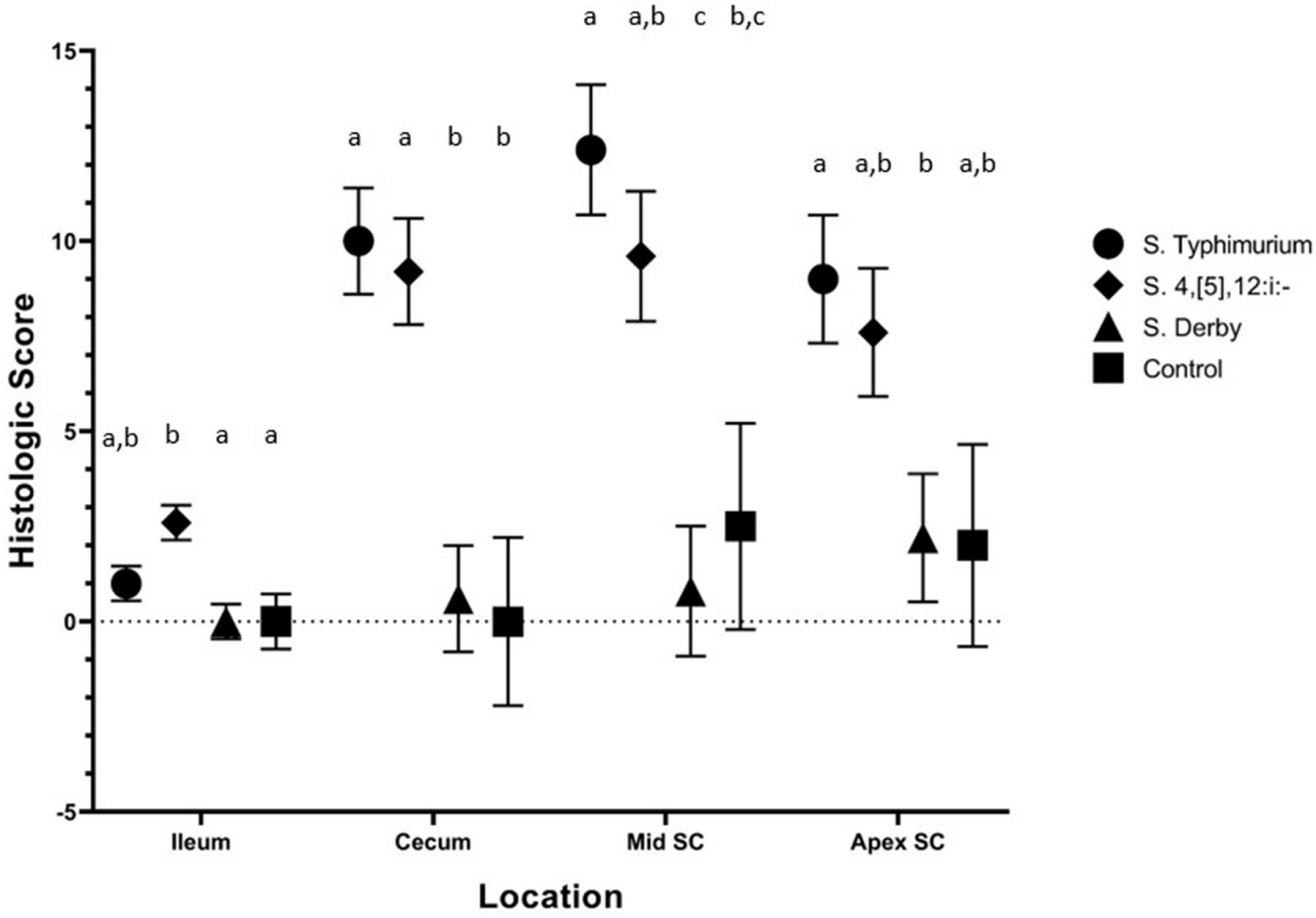
Comparison of histologic lesion scores from pigs inoculated with *Salmonella* serovars 4,[5],12:i:-, Typhimurium, and Derby and non-inoculated control pigs in animal study #2. Histologic lesion scores represent a summary of the ulceration, neutrophil infiltration, crypt elongation and abscessation, and submucosal inflammation at the time of necropsy on DPI 2. Mean and standard error are represented by the symbols and vertical lines, respectively. Different letters indicate statistically significant differences between serovar groups, separated by tissue location (*p* < 0.05). Histologic lesion scores from pigs euthanized on DPI 4 and 28 depicted in Supplemental Figure 2.

Histopathologic lesions observed upon evaluation of tissues from necropsied animals were primarily limited to ulceration, neutrophil infiltration, and crypt elongation in the cecum and spiral colon for all serovars. On DPI 2 (Figure 8), there were statistically significant differences in histologic lesion scores in the ileum, cecum, and spiral colon. In the ileum, *S*. 4,[5],12:i:- had induced significantly higher histologic lesion scores than the sham-inoculation and *S*. Derby but not more severe than *S*. Typhimurium. In the cecum, *S*. Typhimurium and *S*. 4,[5],12:i:- -inoculated pigs had a significantly greater histologic score than control pigs and *S*. Derby-inoculated pigs but not greater than one another. In the mid (or lesion of) spiral colon, *S*. 4,[5],12:i:- had induced significantly different histologic lesion scores from *S*. Derby only while *S*. Typhimurium had induced significantly different histologic lesion scores than *S*. Derby and controls. In the apex of the spiral colon, *S*. Typhimurium-inoculated pigs had a significantly greater histologic score than *S*. Derby-inoculated pigs; however, *S*. 4,[5],12:i:- mean histologic score was not significantly different from those inoculated with *S*. Derby or the control group. On DPI 4 (Supplemental Figure 2A), there were statistically significant differences in the histologic scores in the cecum and mid (or lesion of) spiral colon only. In the cecum, *S*. 4,[5],12:i:- -inoculated pigs had significantly more severe lesions than those inoculated with *S*. Derby and controls; *S*. Typhimurium-inoculated animals did not have a significantly higher histologic score than of *S*. 4,[5],12:i:-, *S*. Derby, or sham-inoculated pigs. In the mid (or lesion of) spiral colon, pigs inoculated with *S*. Typhimurium had significantly higher histologic scores than pigs inoculated with *S*. Derby and control pigs. On DPI 28 (Supplemental Figure 2B), there were no statistically significant differences in the histologic scores between the treatment groups in any of the intestinal sections evaluated. Overall, histopathologic lesions suggestive of clinical salmonellosis were present consistently in the *S*. Typhimurium and *S*. 4,[5],12:i:- -inoculated groups on DPI 2 and 4 but not the *S*. Derby-inoculated or control groups. Thus, *Salmonella* 4,[5],12:i:- infection resulted in a similar severity of diarrhea, disturbance in rectal temperature, colonization of tissues, and gross and histologic lesions as with *Salmonella* Typhimurium infection.

It is worthwhile to mention that other potential secondary swine pathogens were encountered during the study. *Balantidium coli*, which has not been noted to cause disease as a primary pathogen in swine, was noted upon histologic examination in a portion of pigs from each *Salmonella-*inoculated group euthanized on DPI 2 (5/5 of 4,[5],12:i:- pigs; 1/5 of Typhimurium group; 1/5 of Derby group; 0/2 of control group) and DPI 4 (5/5 of 4,[5],12:i:- group, 3/5 of Typhimurium group, 2/5 of Derby group, and 0/3 of control group). By DPI 28, the proportion of pigs with *B. coli* present on histopathologic examination decreased substantially (1/10 of 4,[5],12:i:- group, 2/10 of Typhimurium group, 0/9 of Derby group, and 0/3 of control group). *Cryptosporidium* was also detected on DPI 2 (in 2/5 of 4,[5],12:i:- group, 1/5 of Typhimurium group, 2/5 of Derby group, and 1/2 of control group) and on DPI 4 (in 3/5 of 4,[5],12:i:- group, 1/5 of Typhimurium group, 3/5 of Derby group, and 2/3 of control group). No *Cryptosporidium* was detected on DPI 28. No histopathologic lesions suggestive of clinical disease caused by enteric pathogens of swine other than *Salmonella* were detected during the histopathologic examination.

### Animal Study #3: Evaluation of the Competitive Fitness of *S*. 4,[5],12:i:- When Co-inoculated With *S*. Typhimurium in Swine

A total of 10 of the 12 co-inoculated pigs and all of the singly inoculated pigs completed the study and were included in the final analysis. One of the co-inoculated pigs died on DPI 3 and was submitted to the ISU-VDL for evaluation; it was determined to have died from septicemia as the ileum, colon, liver, spleen, tonsils, and ileocecal lymph nodes were all culture positive for *Salmonella*. One additional pig was euthanized on DPI 6 due to neurologic deficits, severe fever, and deteriorating condition. This pig was also submitted for evaluation at the ISU- VDL, and was determined to have meningoencephalitis caused by *Haemophilus parasuis*. Both of these pigs were removed from the analysis, leaving 10 pigs for analysis in the co-infected group. While the original intent was to utilize the same 4,[5],12:i:- and Typhimuirum isolates in both animal studies #2 and #3, due to an inability to clearly demonstrate differentiation of these isolates using their resistance profiles and selective media, two new isolates of each strain with complementary resistance profiles that could be clearly differentiated on selective media were chosen for this study.

#### Clinical Disease

To ensure that both the *S*. Typhimurium and *S*. 4,[5],12:i:- isolates were able to cause comparable disease when infecting a pig individually, three pigs were inoculated with only *S*. Typhimurium and three with only *S*. 4,[5],12:i:-. In *S*. 4,[5],12:i:- -infected pigs, the mean temperature peaked on DPI 2 at 102.5°F (±0.1) (39.2°C ± 0.1)*. Salmonella* Typhimurium-infected pigs had a peak mean temperature of 102.4°F (±0.5) (39.1°C ± 0.3) on DPI 4. The peak mean fecal scores of each group were 3.7 (±0.3) on DPI 3 in *S*. 4,[5],12:i:- -infected pigs and 3.0 (±0.6) on DPI 2 and DPI 4 in *S*. Typhimurium-infected pigs. These results indicated that both isolates were able to individually cause mild clinical disease in pigs. In pigs that received both *S*. Typhimurium and *S*. 4,[5],12:i:-, the mean fecal score peaked on DPI 1 at 4.0 (±0.3) but remained above 3.5 on DPI 2, 3, and 4. The mean temperature of co-infected pigs peaked at 103.3°F ± 0.3) (39.6°C ± 0.2) on DPI 2. There was a notable increase in the severity and duration of clinical disease in pigs infected simultaneously with *S*. Typhimurium and *S*. 4,[5],12:i:- compared to pigs infected with either serovar of *Salmonella* individually, however, the inoculum dose for the co-infected pigs was double that of the singly-infected animals.

#### Bacterial Culture

**Figure 9:**
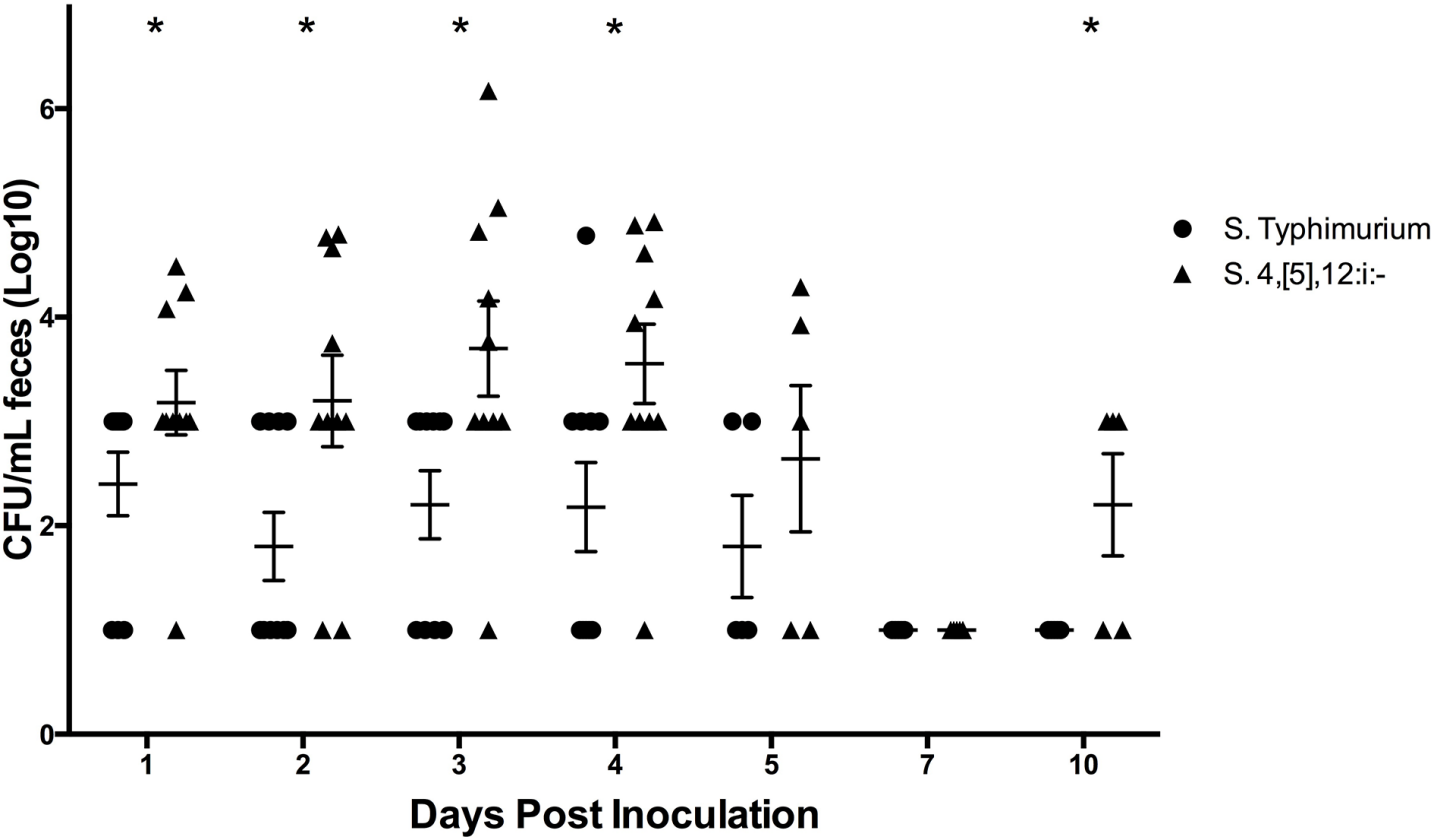
Quantitative fecal culture results from animal study #3 in which pigs were co-inoculated with equal amounts of serovars of *Salmonella*: 4,[5],12:i:- and Typhimurium. Each data point represents the log_10_ CFU/mL result in individual animals. The mean and standard error are represented by the horizontal and vertical lines, respectively. Asterisks represent days in which the mean amount of *S*. 4,[5],12:i:- was significantly different from the mean amount of *S*. Typhimurium (*p* < 0.05).

In the 10 pigs that completed the co-inoculation study, there was a higher mean concentration of *S*. 4,[5],12:i:- detected via culture compared to *S*. Typhimurium at DPI 1, 2, 3, 4, 5, and 10, and the difference was statistically significant on DPI 1, 2, 3, 4, and 10 (Figure 9). The largest difference in amounts of *S*. 4,[5],12:i:- and *S*. Typhimurium occurred on DPI 2. On DPI 7, the only time point in which the average amount of *S*. Typhimurium exceeded the amount of *S*. 4,[5],12:i:- in feces, the difference was not statistically significant. Of the three pigs singly inoculated with *S*. 4,[5],12:i:- and four samples collected from each pig on DPI 1–4, only three out of 12 samples had detectable levels of *Salmonella* present in their feces. Similar results were found with the *S*. Typhimurium pigs, with only three out of 12 samples possessing detectable levels of *Salmonella* (Supplemental Figure 3).

**Figure 10:**
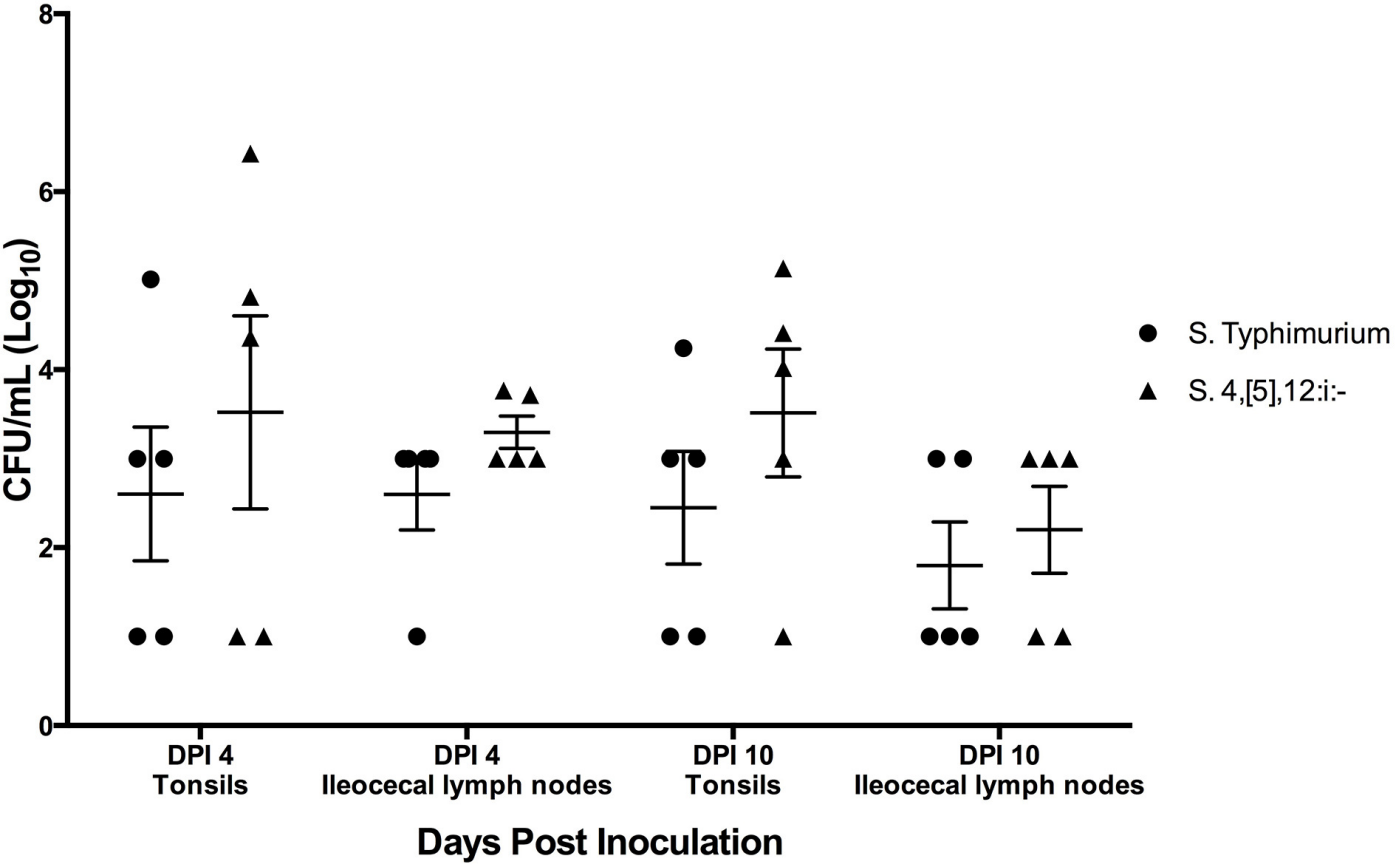
Quantitative culture results from samples collected upon necropsy in animal study #3 in which pigs were simultaneously co-inoculated with equal amounts of two serovars of *Salmonella*: 4,[5],12:i:- and Typhimurium. Each data point represents the log_10_ CFU/mL result in individual animals. The mean and standard error are represented by the horizontal and vertical lines, respectively.

All three pigs singly infected with *S*. Typhimurium were positive for *Salmonella* in their tonsils and ileocecal lymph nodes on DPI 4. Of the three pigs infected singly with *S*. 4,[5],12:i:-, only one had *Salmonella* present in the tonsils on DPI 4, and two had *Salmonella* in the ileocecal lymph nodes (Supplemental Figure 3). Of the pigs that were infected simultaneously with both *S*. Typhimurium and *S*. 4,[5],12:i:-, seven of the 10 pigs had tonsils culture positive for *S*. 4,[5],12:i:- and six pigs of the 10 were positive for *S*. Typhimurium (Figure 10). Of those pigs positive for *Salmonella* in their tonsils, five had higher levels of *S*. 4,[5],12:i:-, two had higher levels of *S*. Typhimurium, and three did not have any detectable *Salmonella*. As for the ileocecal lymph nodes, eight pigs were positive for *S*. 4,[5],12:i:- and six were positive for *S*. Typhimurium. Similar to the tonsils, five had higher levels of *S*. 4,[5],12:i:-, one had higher levels of *S*. Typhimurium, four had equivalent levels of the two serovars, and one did not have any detectable *Salmonella*.

#### Competition Index

**Figure 11:**
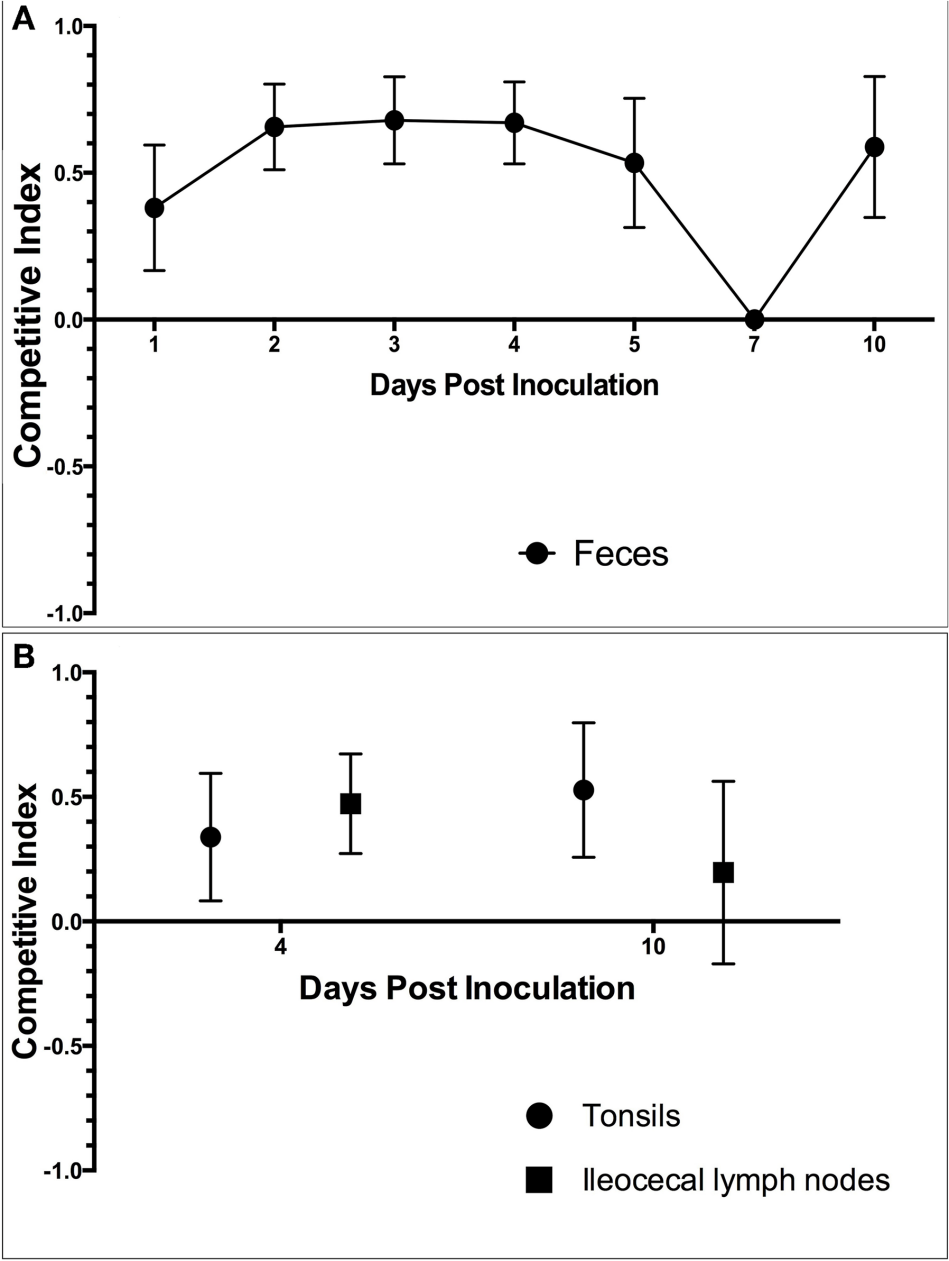
The competition index (CI) calculated based on the culture results from animal study #3. The CI was calculated for **(A)** fecal samples collected throughout the study period and **(B)** samples collected at the time of necropsy on DPI 4 and 10. The CI represents the fitness of *Salmonella* 4,[5],12:i:- relative to *Salmonella* Typhimurium, with a positive CI indicating that *S*. 4,[5],12:i:- is more fit within the host and a negative CI indicating that *S*. Typhimurium is more fit within the host. Values closer to 1 or −1 indicates that 4,[5],12:i:- or Typhimurium were the dominant *Salmonella* serovar in that set of samples, respectively.

The competition index (CI) was calculated for each set of samples collected, including feces, tonsils and lymph nodes. In the feces on all days except for DPI 7, the competition index was greater than zero, indicating that *S*. 4,[5],12:i:- had a higher level of fitness in relation to intestinal colonization compared to *S*. Typhimurium (Figure 11A). The CI was also greater than zero in both the tonsils (0.32 and 0.52) and ileocecal lymph nodes (0.39 and 0.2) on DPI 4 and DPI 10 (Figure 11B), respectively. These results indicate that *S*. 4,[5],12:i:- also demonstrated a higher level of fitness in colonization of tonsils and ileocecal lymph nodes when compared to *S*. Typhimurium.

### Whole Genome Sequencing Analysis of the *Salmonella* Isolates Utilized in Animal Studies #2 and #3

The sequencing results of the five isolates utilized in animal studies #2 and #3 were analyzed for MLST, plasmid type, virulence gene presence and antimicrobial resistance gene presence. Supplemental Table 2 includes the results of the MLST profile, with both *S*. 4,[5],12:i:- isolates belonging to the ST34 clade, both *S*. Typhimurium isolates belonging to the ST19 clade, and the Derby isolate belonging to the ST40 clade. PlasmidFinder was used to identify the presence of plasmid replicons in the 5 strains used in the study (Supplemental Table 3). All isolates of *Salmonella* utilized in these studies harbored at least 2 plasmid replicons. Plasmid replicons were all unique between each isolate, except for the identification of IncI1 in both the *S*. Derby isolate and one of the *S*. typhimurium isolates. Virulence Factor Database was used to identify the presence of known pathogenicity genes in *Salmonella* (summary Table 4; detailed Supplemental Table 4). Overall, the profile of virulence genes was very similar for the four *S*. 4,[5],12:i:- and *S*. Typhimurium isolates; however, several genes including *lpf, stc, stj*, and *sodC1* were notably absent in the *S*. Derby isolate. Conversely, the *S*. Derby isolate harbored several virulence operons not present in either of the four *S*. 4,[5],12:i:- and *S*. Typhimurium isolates, including *peg, sta*, and *ste*. Finally, ARG-Annot was utilized to identify the antimicrobial resistance genes present in the isolates (Supplemental Table 5); this data was then compared to the phenotypic data obtained via the initial clinical diagnostic workup when the isolate was identified. Both sequenced *S*. 4,[5],12:i:- isolates carried the previously described genotypic profile for ampicillin, streptomycin, sulfonamides, and tetracycline resistance (referred to as ASSuT) that has been defined to include the simultaneous presence of *blaTEM-1, strA, strB, sulII, and tet(B)* genes (48, 49). Additional resistance genes including *bla*_CMY_ (ISU-244-16 only) and *qnrB* (ISU-240-15 only), which are commonly associated with plasmids, also were present and conferred resistance to cephalosporins and fluoroquinolones, respectively.

**Table 4:** Results of Virulence Factor Database (VFDB) analysis for the presence of known virulence genes based on whole genome sequencing of isolates of *Salmonella* utilized for inoculation of pigs in animal studies #2 and #3.

			**Derby**	**4,[5],12:i:-**		**Typhimurium**	
**VFclass**	**Virulence factors**	**Total number of related genes**	**ISU-SAL242-16**	**ISU-SAL240-15**	**ISU-SAL245-16**	**ISU-SAL243-14**	**ISU-SAL244-16**
Capsule	Vi antigen (Tvi/Vex)	10	0	0	0	0	0
Fimbrial adherence determinants	Agf/Csg	7	7	7	7	7	7
	Bcf	7	7	7	7	7	7
	Fim	9	9	9	9	9	9
	Lpf	5	0	5	5	0	5
	Pef	4	0	0	0	4	0
	Peg	4	4	0	0	0	0
	Saf	4	4	4	4	4	4
	Sef	4	0	0	0	0	0
	Sta	7	7	0	0	0	0
	Stb	5	5	5	5	5	5
	Stc	4	0	4	4	4	4
	Std	3	3	3	3	3	3
	Ste	6	6	0	0	0	0
	Stf	5	5	5	5	5	5
	Stg	4	0	0	0	0	0
	Sth	4	4	4	4	4	4
	Sti	4	4	4	4	4	4
	Stj	5	0	5	5	5	5
	Stk	7	0	0	0	0	0
	Tcf	4	0	0	0	0	0
Macrophage inducible genes	Mig	1	1	1	1	1	1
Magnesium uptake	Mgt	2	2	2	2	2	2
Non-fimbrial adherence determinants	MisL	1	1	1	1	1	1
	RatB	1	1	1	1	1	1
	ShdA	1	1	1	1	1	1
	SinH	1	1	1	1	1	1
Regulation	PhoPQ	2	2	2	2	2	2
Secretion system	TTSS (SPI-1 encode)	30	30	30	30	30	30
	TTSS (SPI-2 encode)	28	28	28	28	28	28
	TTSS−1 and−2 translocated effectors	2	1	1	1	1	1
	TTSS-1 translocated effectors	10	10	10	9	9	10
	TTSS-2 translocated effectors	15	15	15	15	14	13
Serum resistance	Rck	1	0	0	0	1	0
Spv locus	Spv	5	0	0	0	5	0
Stress adaptation	SodCI	1	0	1	1	1	1
Toxin	Typhoid toxin	3	0	0	0	0	0

*Green shading indicates that all genes were present for that specific virulence factor; yellow indicates most but not all genes were present*.

## Discussion

The work completed in these studies clearly demonstrates that for the representative isolates selected, *Salmonella* serovar 4,[5],12:i:- possessed a comparable ability to *Salmonella* Typhimurium to cause significant clinical disease in swine. In addition, this serovar, along with Typhimurium and Derby, can be carried in the tonsils and lymph nodes and shed in the feces of infected animals for weeks following exposure and illness. Potential sequelae to this carriage includes likely contamination of the environment with subsequent infection of pen mates and contamination of carcasses and meat at harvest leading to food safety concerns. Overall, all three animal studies provided insight into the pathogenesis of disease caused by *S*. 4,[5],12:i:- in swine. Animal study #1 allowed us to compare three separate isolates of *S*. 4,[5],12:i:-, all of which had retained their ability to cause disease through the culture process as indicated by the observed fever and/or diarrhea as well as histopathologic evidence of disease. Fecal shedding for all isolates continued for the duration of the study from DPI 1–7. Animal study #2 enabled evaluation of the pathogenicity, fecal shedding, and colonization of swine by *S*. Typhimurium, *S*. 4,[5],12:i:-, and *S*. Derby. The mean rectal temperature of pigs infected with all three *Salmonella* serovars peaked at DPI 1–2. The mean fecal score reached its peak at DPI 2 for *S*. Typhimurium and *S*. 4,[5],12:i:- -inoculated pigs, with *S*. Derby-inoculated pigs reaching a lower peak at a later time than the other two serovars. The rectal temperatures and fecal scores indicated that *S*. 4,[5],12:i:- has a similar disease-causing ability to *S*. Typhimurium and induces a more severe disease than that caused by *S*. Derby. Detectable fecal shedding continued through DPI 28 for *S*. Typhimurium-innoculated pigs and DPI 21 for *S*. 4,[5],12:i:- -inoculated pigs and *S*. Derby-inoculated pigs. The colonization of tonsils and ileocecal lymph nodes appeared to be similar across DPI 2, 4, and 28 in all three serovar groups. Gross lesions suggestive of salmonellosis were primarily observed on DPI 2 and 4 and were limited to those pigs infected with *S*. Typhimurium or *S*. 4,[5],12:i:-. Histologic lesions occurred primarily in the cecum and spiral colon, with significantly higher scores in the pigs infected with *S*. Typhimurium and *S*. 4,[5],12:i:- as compared to *S*. Derby. Finally, animal study #3 revealed that for the isolates selected, *S*. 4,[5],12:i:- demonstrated a competitive advantage over *S*. Typhimurium *in vivo*, which was evidenced by the significantly higher mean levels of *S*. 4,[5],12:i:- cultured from various tissues and feces relative to *S*. Typhimurium on DPI 1, 2, 3, 4, and 10.

Many studies have demonstrated that *S*. 4,[5],12:i:- is a monophasic variant of *S*. Typhimurium, however, the characterization of *S*. 4,[5],12:i:- as a pathogen of swine has not been as readily documented. To our knowledge, only two studies have been published on experimental infections of swine with *S*. 4,[5],12:i:-, leaving a large gap in our understanding of this emerging serovar. One of the recently published studies was a small-scale study in experimentally infected swine, which revealed that *S*. 4,[5],12:i:- has maintained a similar disease-causing ability to that typically associated with *S*. Typhimurium (21). Specifically, at DPI 2, the infected pigs developed a fever and diarrhea, and they shed *S*. 4,[5],12:i:- in their feces throughout the 7 days following inoculation (21). Overall, the authors of that study concluded that both *S*. Typhimurium and its monophasic variant, *S*. 4,[5],12:i:-, can cause gastrointestinal disturbances, further substantiating the claim that expression of only one flagellar phase does not alter the pathogenicity (21, 34). The results of this small-scale study with respect to the clinical disease established by *S*. 4,[5],12:i:- was in alignment with our work as we determined that diarrhea and fever occur on DPI 2 following infection with *S*. 4,[5],12:i:- and that the disease and lesions induced by *S*. 4,[5],12:i:- closely resemble that induced by *S*. Typhimurium. However, that study utilized fecal moisture content to determine if the pigs had developed diarrhea (21). Fecal moisture content analysis is a much more objective way to evaluate the level of diarrhea in swine compared to fecal scoring, even though fecal scoring is a more common method in studies of this type (41, 50). Due to the size and scope of our study, we utilized fecal scoring to facilitate evaluation of a larger sample size.

The second and more recent study was similar to our comparison of *Salmonella* serovars 4,[5],12:i:-, Typhimurium, and Derby but did not include a control group for comparison (20). In contrast to our findings, this study found that all *S*. 4,[5],12:i:-, *S*. Derby, and *S*. Typhimurium-inoculated pigs, with the exception of one *S*. Derby-inoculated pig on DPI 21, one *S*. Typhimurium-inoculated pig on DPI 14, and one *S*. Typhimurium-inoculated pig on DPI 45, were shedding *Salmonella* throughout the study at all time points from DPI 1-49 (20). This is contrary to the findings of our study and other studies that have reported that *Salmonella* shedding varies on an individual basis, varies with the infecting serovar, and is not continuous (2, 25, 26, 28). The deviation in this study from what has been reported previously from other studies and our findings is likely due to the larger amount of feces collected for culture, as this study used 30 grams of feces as opposed to approximately 0.5 grams of feces used in our study and an increased fecal sample mass has been correlated to increased analytical sensitivity (51). This study also reported that *S*. 4,[5],12:i:- infection of swine resulted in fever and diarrhea on DPI 21 while diarrhea observed in pigs infected with *S*. Typhimurium occurred on DPI 7 and 10 and with *S*. Derby on DPI 14 (20). This is much different from that observed in other studies of experimental infections with *S*. Typhimurium and *S*. 4,[5],12:i:- in swine, which generally report fever and diarrhea on DPI 2–4, as well as our study which also indicated disease occurs much sooner after infection. As is the case for selection of bacterial isolates for use in any animal study, there is the potential that variation in pathogenicity between isolates of the same bacterial species or serovar can exist and may complicate comparisons between studies.

*Salmonella* Typhimurium is well-recognized as an enteric pathogen of swine. Many studies have been completed to determine the course of disease typical of the pathogen. It caused significantly increased rectal temperatures and fecal scores in 3 to 4 week-old pigs at DPI 2–3 while also colonizing the liver, spleen, tonsils, and ileocecal junction (52). Another study echoed similar findings in 4 week-old experimentally infected pigs, with 100% of pigs shedding *Salmonella* in their feces through DPI 28, 20% developing a fever and 55% exhibiting diarrhea (53). Both of these studies had similar conclusions to ours from animal study #2 in that *S*. Typhimurium causes fever, diarrhea, and fecal shedding of the organism following infection. *Salmonella* Derby has been less thoroughly evaluated *in vivo* in swine, likely due to its lesser-pathogenicity. However, one study found that it is shed in the feces of some pigs through DPI 56 and potentially even longer, indicating an ability to persist within the host (28). The results from our study showed *S*. Derby is shed for a shorter duration in the feces, specifically through DPI 21, compared to the study that reported fecal shedding of *S*. Derby occurs for 8 weeks post infection. However, given the presence of *Salmonella* in the tissues collected on DPI 28 from all three groups, including tissues from pigs inoculated with *S*. Derby, it is clear that all of these serovars of *Salmonella* are able to persist within exposed pigs through DPI 28. The absence of *Salmonella* in feces was likely the result of low diagnostic sensitivity in our study and/or a low quantity of *Salmonella* being shed rather than an absence of sustained infection.

A subset of pigs in animal study #2 developed a fever while another subset developed hypothermia. When comparing the mean rectal temperatures of the pigs in each treatment group, it was noted that *S*. 4,[5],12:i:- did not appear to successfully induce a fever. However, evaluation of the rectal temperatures of the pigs by the percentage within, above, or below the normal temperature range (101.5–103.5°F or 38.6–39.7°C) revealed that *S*. 4,[5],12:i:- caused fever in some and hypothermia in others. Specifically, in the first 4 days following infection, *S*. 4,[5],12:i:- had caused 25–90% of pigs to have rectal temperatures outside of the normal range, which was comparable to *S*. Derby-infected pigs with 11–50% outside of the normal range and *S*. Typhimurium-infected pigs with 20–35% outside of the normal range. This is in contrast to the control group in which no more than 22% of pigs had rectal temperatures outside of the normal range during the same timeframe. Fever is widely accepted as a response to infection, as it creates a more hostile environment for the bacteria within the host to improve host resistance to spread of the infection. However, hypothermia represents another potential response to infection. Hypothermia is a mechanism in place thought to downregulate pro-inflammatory cytokine release to reduce excessive tissue damage and is generally associated with severe systemic infection (54, 55). Thus, calculation of the average temperature in each group may not be the best measure of clinical disease manifestation in these groups as severely ill animals can also exhibit lower than normal body temperatures. Estimation of total number of animals with temperatures outside the normal range might represent a better indication of clinical disease in this case. For example, on DPI 1, 2, 3, and 4, 25, 45, 27, and 87%, respectively, of *S*. 4,[5],12:i:- -infected pigs had rectal temperatures outside the normal range of 101.5–103.5°F (38.6–39.7°C). This is in contrast to 0, 18, 0, and 22% for control pigs over the same time period. Oddly, the control pigs as well as the *S*. 4,[5],12:i:- -infected pigs also exhibited as a group decreased body temperatures between DPI 5–7; as the whole group experienced this change, it is likely that room temperature fluctuations related to normal husbandry activities such as cleaning of the pens contributed to this changes in these groups. All four experimental groups were housed in separate rooms throughout the study, which may explain why this was not seen in the other two groups.

When co-inoculated at the same levels, *S*. 4,[5],12:i:- was consistently detected in the feces of a higher percentage of pigs and at higher levels than *S*. Typhimurium in our study. Additionally, the competition index within the feces, tonsils and ileocecal lymph nodes demonstrated that *S*. 4,[5],12:i:- has a greater level of fitness in colonization of the host than *S*. Typhimurium. While this study only represents a comparison of a single isolate each of *S*. 4,[5],12:i:- and *S*. Typhimurium and further studies with multiple isolates are warranted, this finding may provide an hypothesis as to why *S*. 4,[5],12:i:- has been increasingly identified in swine diagnostic samples over the past several years. Although no studies have been published on the clinical disease resulting from simultaneous infections with more than one serovar of *Salmonella*, there have been reports of simultaneous infections of *Salmonella* and other viral or bacterial pathogens of swine having additive effects (56, 57). Accurate identification of co-infections is likely rare in clinical practice as only a single colony of *Salmonella* is typically selected from the culture plate for final identification and characterization, leaving the possibility for co-infections to be occurring frequently yet rarely detected. Future research is warranted to better understand the impact that co-infections might play in clinical disease in the field. Our results suggest the potential for synergism between *S*. 4,[5],12:i:- and *S*. Typhimurium when present simultaneously in swine, but a larger sample size and equivalent inoculum concentrations between co-infected and singly-infected groups would be necessary to confirm this finding. As all pigs were inoculated with the same dose of each serotype, this resulted in double the inoculation dose for the co-infected pigs when compared to the singly-inoculated groups. While this is a comparatively small difference when compared to a logarithmic scale, if this study were to be repeated, administration of the same total dose of *Salmonella* in the inoculum for singly and co-infected pigs would aid in differentiation of the effect of the dose from the effect of the co-infection.

At the time the co-inoculation study was conducted, whole genome sequencing of the isolates had not yet been performed. Further investigation of the genotypic antimicrobial resistance genes present in the isolates suggests that the resistance to gentamicin in the *S*. 4,[5],12:i:- isolate was likely mediated through the *strA/strB* genes which have been shown to be frequently inserted into the chromosome in *S*. 4,[5],12:i:- ASSuT resistant isolates (48, 49). Resistance to ceftiofur in the *S*. Typhimurium isolate was likely mediated by *bla*_CMY_, which has been previously associated with the IncI1 plasmid which was also identified via whole genome sequencing in this isolate (58). In theory it is possible that transfer of these AMR genes may have occurred between our isolates *in vivo*, however, further investigation would be necessary to definitively prove that the *bla*_CMY_ gene was indeed located on this plasmid and determine if conjugative transfer genes such as *tra* or *pil* were present on same plasmid which would make the possibility of transfer more likely (58). Of the two resistance genotypes utilized for differentiation in this study, it is much more likely that exchange of plasmid-associated genes such as *bla*_CMY_ could have occurred rather than exchange of genes believed to be chromosomally-located. If this had occurred in high numbers, we would have expected that the culture results for the *S*. Typhimurium isolate may have appeared elevated compared to the *S*. 4,[5],12:i:- isolate regardless of which serovar was actually more successful in colonizing the host. This was not the case, and screening of colonies via serovar specific rtPCR also did not identify any colonies that were not of the appropriate serovar present on the opposite selective plate. Therefore, while gene transfer is theoretically possible, it does not appear likely that this occurred in high enough numbers to affect the results of the study.

Both isolates of *S*. 4,[5],12:i:- utilized in animal studies #2 and #3 belonged to the ST34 sequence type, harbored the ASSuT resistance profile, and are likely part of a recently emerged clade of *S*. 4,[5],12:i:- that appears to be the predominate clade in swine production in the United States (59). The *S*. Typhimurium isolates belonged to the ST19 sequence type which has been circulating in the Midwest since 2000 (59); this provides additional evidence that suggests that the *S*. 4,[5],12:i:- isolates selected did not evolve from local *S*. Typhimurium but instead are more similar to a multi-drug resistant clade first reported in Europe (10). Based on the commonality between previously described circulating strains of *S*. 4,[5],12:i:- and *S*. Typhimurium, it is reasonable to suggest that the pathogenicity results presented here are representative of the circulating *S*. 4,[5],12:i:- and *S*. Typhimurium isolates within swine production systems in the Midwest United States today. Both isolates of *S*. 4,[5],12:i:- contained similar virulence genes to that of the *S*. Typhimurium isolates, but differed significantly from the *S*. Derby isolate. This difference was particularly noticeable within the fimbrial adherence determinates (Lpf, Stc and Stj were present only in *S*. 4,[5],12:i:- and *S*. Typhimurium; Peg, Sta, and Ste were present only in *S*. Derby). It is plausible that differences in the determinates of the ability to adhere within the gastrointestinal tract might help explain some of the differences observed in pathogenicity within the swine host. However, several genes known to be involved in virulence, including *sipC* which is involved in cell adhesion and invasion, *sopB* which promotes the influx of inflammatory cells and fluid secretion involved in diarrhea, and *hilA* which activates the invasion process (37), were found to be present in all isolates sequenced including *S*. Derby.

A recent study attempted to determine the pathogenicity potential of various isolates of *Salmonella* in humans by comparing whole genome sequencing and the presence of known virulence genes to a phenotypic assessment of *in vitro* virulence (60). Within that study, *S*. 4,[5],12:i:- was considered to have a high probability of *in vitro* infection [P (inf)], similar to but slightly less than that of *S*. Typhimurium, which had the highest probability of all isolates studied. Unfortunately, the variability in P (inf) between individual strains tested was greater than that of the variability between each serovar, therefore, they could not rank the probability of *in vitro* infection based on serovar alone. The presence of plasmid-associated virulence genes (*perf, rck*, and *spv)* was associated with increased P (inf) and was present in all but one *S*. Typhimurium strain studied, but none of the *S*. 4,[5],12:i:- isolates despite both strains having high P (Inf). In our study, only one of the *S*. Typhimurium isolates (ISU-SAL243-14) displayed these genes. The *sodC1* gene, however, was present in the *S*. Typhimurium and *S*. 4,[5],12:i:- strains tested but absent in *S*. Derby; this gene was associated with high P (inf) and has been previously described to be present in *S*. 4,[5],12:i:- and *S*. Typhimurium (60). That study focused on the genetic determinates of disease in humans and it is unclear how transferrable these results are to swine, therefore, further work is needed to better understand the genetic determinates of virulence within the swine host.

Our studies were not completed without limitations. All samples collected for culture were frozen at −80°C from the time of collection to the time of processing up to 2 months later. The freezing and thawing process may have reduced the viability of some of the *Salmonella* initially present in the samples (61). Ideally, all samples would have been processed within several hours of collection without any freezing; unfortunately, the large volume of samples collected at any given time point made this option less feasible. An alternative to culture is PCR testing. Many PCR tests have been validated for the detection and identification of *Salmonella*, which would have also been able to quantify the *Salmonella* present in the samples, however, this method was not cost-effective for the large number of samples collected during this study. An additional culture-related limitation was the relatively small amount of feces (~0.5 grams) used for culture. The diarrhea induced by the *Salmonella* infection created challenges regarding collection of large amounts of feces from the rectum of each pig, therefore, a lesser amount of starting material was used than might have been ideal to increase the sensitivity of the test.

In an effort to increase the odds of detecting acute gross and histologic lesions induced by *Salmonella* infections, *Salmonella*-infected pigs in animal study #2 were selected for euthanasia on DPI 2 and 4 based on severity of clinical disease. That is, pigs with the highest combined fecal score and rectal temperature in each pen were selected for euthanasia. There is known to be significant individual-level variation in the effects of *Salmonella* infections in pigs, with some pigs even failing to develop clinical signs and/or gross lesions suggestive of infection (29, 62). If there is a correlation between the severity of clinical disease and persistence of infection or colonization of tissues, these measurements could be biased. The method of selection utilized on DPI 2 and 4 likely also increased the mean gross and histologic lesion scores on these days while potentially decreasing the mean scores on DPI 28. It also may have decreased the mortality rate following *Salmonella* infections and altered the average temperature and fecal scores of the groups following each euthanasia time point. However, it enabled characterization of the location and types of lesions induced by *Salmonella*.

Finally, despite thoroughly screening all animals for diseases of specific concern prior to enrollment in the studies, there were a few health issues identified in the animals during the studies that have the potential to confound the results. In an attempt to decrease the affect of these issues, two animals each were not included in the final analysis for both animal studies #2 and #3. In addition, the fecal scores of the control and *S*. 4,[5],12:i:- groups were mildly increased prior to initation of animal study #2, which could have been due to infection by a non-*Salmonella* pathogen. Further testing of samples for other pathogens was not pursued given the mild nature of the observed changes and lack of gross or histopathologic evidence of disease caused by other pathogens of concern upon necropsy. During the histopathologic evaluation of tissues from animal study #2, *Cryptosporidium* and *Balantidium coli* were also observed in a subset of the pigs in all serovar groups and the control group at all euthanasia time points. The presence of *Cryptosporidium* did not appear to cause a severe increase in the fecal scores, rectal temperatures, or histopathologic scores in any of the treatment groups. This is evidenced by the fact that in a comparison of the mean fecal scores, rectal temperatures, and histopathologic scores, the majority of measurements were either equivalent between pigs that were positive and negative for *Cryptosporidium* or higher in those that were negative for *Cryptosporidium*. *Cryptosporidium* can cause a mild and self-limiting diarrhea in pigs, although it generally causes an asymptomatic infection, so its effect on the pigs in this study remains unknown. *Cryptosporidium* was not one of the pathogens that the pigs were screened for prior to arrival, so it is unclear when the infection was obtained. While it would be most ideal to screen the pigs for all possible pathogens including *Cryptosporidium* prior to enrollment in the study, it would be an inefficient use of resources and was not practical for this study; therefore, the pathogens of the highest concern were selected for screening. *Balantidium coli* was also noted in the intestinal sections of some pigs. However, this is not generally considered to be a primary pathogen and was likely not independently the cause of diarrhea in the pigs (63). As neither of the previous studies evaluating the pathogenicity of *S*. 4,[5],12:i:- reported histopathologic results, nor have the majority of studies evaluating the pathogenicity of various *Salmonella* serovars in swine, these organisms may be frequently present in studies of this nature in this age pig yet be frequently not reported. In addition, while it cannot be claimed that our control group was an ideal control as there were temperature and fecal score variations within this group throughout the study, again, neither of the previous studies evaluating the pathogenicity of *S*. 4,[5],12:i:- contained a control group for comparison.

## Conclusion

In conclusion, our results clearly indicate that for the chosen isolates, *S*. 4,[5],12:i:- induces clinical disease comparable to that of *S*. Typhimurium with similar corresponding gross and histopathologic lesions. The cause of the emergence of the monophasic serovar may be due in part to a competitive advantage *S*. 4,[5],12:i:- possess *in vivo*, as evidenced by the higher mean levels of *S*. 4,[5],12:i:- relative to *S*. Typhimurium in a majority of pigs infected with the two serovars simultaneously. Future research should focus on assessing the frequency and potential synergistic effects of concurrent *S*. 4,[5],12:i:- and *S*. Typhimurium infections in swine as well as further investigation into the rapid emergence of *S*. 4,[5],12:i:- in swine production in the U.S.

## Data Availability Statement

The datasets generated for this study can be found in the NCBI Database under BioProject ID number PRJNA575700 (http://www.ncbi.nlm.nih.gov/bioproject/575700).

## Ethics Statement

The animal study was reviewed and approved by Iowa State University Institutional Animal Care and Use Committee (IACUC) - approval #11-16-8391-S.

## Author Contributions

AJK, ACK, BA, OS, KS, and EB were responsible for the initial design of the study. SN was responsible for performing the experimental studies with assistance from AJK, BA, PA, EB, DM, FM, IG, and HM. BA, PA, KS, and EB were responsible for necropsy and sample collection. Data analysis was performed by SN and AJK, with statistical analysis performed by CW and SN. All authors reviewed and approved the manuscript prior to submission.

### Conflict of Interest

The authors declare that the research was conducted in the absence of any commercial or financial relationships that could be construed as a potential conflict of interest.

## References

[1] Fedorka-Cray PJ Gray JT Wray C *Salmonella* infections in pigs. In: Wray C Wray A, editors. Salmonella in Domestic Animals (New York, NY: CABI Publishing) (2000). p. 191–208.

[2] Nair S Farzan A O'Sullivan TL Friendship RM. Time course of *Salmonella* shedding and antibody response in naturally infected pigs during grower-finisher stage. Can J Vet Res. (2018) 82:139–45. 29755194 PMC5914076

[3] Farzan A Friendship RM. A clinical field trial to evaluate the efficacy of vaccination in controlling *Salmonella* infection and the association of *Salmonella*-shedding and weight gain in pigs. Can J Vet Res. (2010) 74:258–63. 21197225 PMC2949338

[4] CDC National Antimicrobial Resistance Monitoring System for Enteric Bacteria (NARMS): Human Isolates Final Report, 2013. Atlanta, GA: U.S. Department of Health and Human Services, CDC (2015).

[5] CDC National Antimicrobial Resistance Monitoring for Enteric Bacteria (NARMS): Human Isolates Final Report, 2014. Atlanta, GA: U.S. Department of Health and Human Services, CDC (2016).

[6] Hong S Rovira A Davies P Ahlstrom C Muellner P Rendahl A. Serotypes and antimicrobial resistance in *Salmonella enterica* recovered from clinical samples from cattle and swine in Minnesota, 2006 to 2015. PLoS ONE. (2016) 11:20. 10.1371/journal.pone.0168016 27936204 PMC5148076

[7] Yuan C Krull A Wang C Erdman M Fedorka-Cray PJ Logue CM. Changes in the prevalence of *Salmonella* serovars associated swine production and correlations of avian, bovine, and swine-associated serovars with human-associated serovars in the United States (1997-2015). Zoonoses Public Health. (2018) 65:648–61. 10.1111/zph.12473 29687621

[8] Naberhaus SA Krull AC Bradner LK Harmon KM Arruda P Arruda BL Emergence of *Salmonella enteric*a serovar 4,[5],12:i:- as the primary serovar identified from swine clinical samples and development of a multiplex real-time PCR for improved *Salmonella* serovar-level identification. J Vet Diagn Investig. (2019) 31:818–27. 10.1177/1040638719883843 31646949 PMC6900717

[9] Echeita MA Herrera S Usera MA. Atypical, fljB-negative *Salmonella enterica* subsp *enterica* strain of serovar 4,5,12:i: appears to be a monophasic variant of serovar typhimurium. J Clin Microbiol. (2001) 39:2981–3. 10.1128/JCM.39.8.2981-2983.2001 11474028 PMC88275

[10] de la Torre E Zapata D Tello M Mejia W Frias N Pena FJG. Several *Salmonella enterica* subsp enterica serotype 4,5,12:i: - phage types isolated from swine samples originate from serotype typhimurium DT U302. J Clin Microbiol. (2003) 41:2395–400. 10.1128/JCM.41.6.2395-2400.2003 12791855 PMC156524

[11] Bugarel M Vignaud ML Moury F Fach P Brisabois A. Molecular identification in monophasic and nonmotile variants of *Salmonella enterica* serovar Typhimurium. Microbiologyopen. (2012) 1:481–9. 10.1002/mbo3.39 23233427 PMC3535392

[12] Boland C Bertrand S Mattheus W Dierick K Wattiau P. Molecular typing of monophasic *Salmonella* 4, 5:i:- strains isolated in Belgium (2008-2011). Vet Microbiol. (2014) 168:447–50. 10.1016/j.vetmic.2013.11.040 24398228

[13] Huoy L Pornruangwong S Pulsrikarn C Chaturongakul S. Molecular characterization of Thai *Salmonella enterica* serotype typhimurium and serotype 4,5,12:i:- reveals distinct genetic deletion patterns. Foodborne Pathog Dis. (2014) 11:589–92. 10.1089/fpd.2013.1723 24906076

[14] Barco L Barrucci F Cortini E Ramon E Olsen JE Luzzi I. Ascertaining the relationship between *Salmonella* typhimurium and *Salmonella* 4,5,12:i:- by MLVA and inferring the sources of human salmonellosis due to the two serovars in Italy. Front Microbiol. (2015) 6:10. 10.3389/fmicb.2015.00301 25983720 PMC4415582

[15] He XH Xu XB Li K Liu B Yue TL Identification of *Salmonella enterica* Typhimurium and variants using a novel multiplex PCR assay. Food Control. (2016) 65:152–9. 10.1016/j.foodcont.2016.01.015

[16] Mastrorilli E Pietrucci D Barco L Ammendola S Petrin S Longo A. A comparative genomic analysis provides novel insights into the ecological success of the monophasic *Salmonella* serovar 4,5,12:i. Front Microbiol. (2018) 9:18. 10.3389/fmicb.2018.00715 29719530 PMC5913373

[17] Yu YM Zeng H Lyons S Carlson A Merlin D Neish AS. TLR5-mediated activation of p38 MAPK regulates epithelial IL-8 expression via posttranscriptional mechanism. Am J Physiol Gastrointest Liver Physiol. (2003) 285:G282–90. 10.1152/ajpgi.00503.2002 12702497

[18] Tallant T Deb A Kar N Lupica J de Veer MJ DiDonato JA. Flagellin acting via TLR5 is the major-activator of key signaling pathways leading to NF-kappa B and proinflammatory gene program activation in intestinal epithelial cells. BMC Microbiol. (2004) 4:24. 10.1186/1471-2180-4-33 15324458 PMC516440

[19] Misselwitz B Barrett N Kreibich S Vonaesch P Andritschke D Rout S. Near surface swimming of *Salmonella* Typhimurium explains target-site selection and cooperative invasion. PLoS Pathog. (2012) 8:19. 10.1371/journal.ppat.1002810 22911370 PMC3406100

[20] Cevallos-Almeida M Martin L Houdayer C Rose V Guionnet JM Paboeuf F. Experimental infection of pigs by *Salmonella* Derby, S. Typhimurium and monophasic variant of *S* Typhimurium: Comparison of colonization and serology. Vet Microbiol. (2019) 231:147–53. 10.1016/j.vetmic.2019.03.003 30955802

[21] Shippy D Bearson B Holman D Brunelle B Allen H Bearson S Porcine response to a multidrug-resistant *Salmonella enterica* serovar I 4,[5],12:i:- outbreak isolate. Foodborne Pathog Dis. (2018) 15:253–61. 10.1089/fpd.2017.2378 29412766

[22] Arruda B Burrough E Schwartz K *Salmonella enterica* I 4,[5],12:i:- associated with lesions typical of swine enteric salmonellosis. Emerg Infect Dis. (2019) 25:1377–9. 10.3201/eid2507.181453 31211677 PMC6590737

[23] Matiasovic J Stepanova H Kudlackova H Havlickova H Sisak F Rychlik I. Immune response of pigs to *Salmonella enterica* serovar Derby and Typhimurium infections. Vet Microbiol. (2014) 170:284–90. 10.1016/j.vetmic.2014.02.003 24613290

[24] Fedorka-Cray PJ Whipp SC Isaacson RE Nord N Lager K. Transmission of *Salmonella* typhimurium to swine. Vet Microbiol. (1994) 41:333–44. 10.1016/0378-1135(94)90029-9 7801533

[25] Ivanek R Osterberg J Gautam R Lewerin SS. *Salmonella* fecal shedding and immune responses are dose- and serotype- dependent in pigs. PLoS ONE. (2012) 7:e34660. 10.1371/journal.pone.0034660 22523553 PMC3327719

[26] Knetter SM Bearson SMD Huang TH Kurkiewicz D Schroyen M Nettleton D. *Salmonella enterica* serovar Typhimurium-infected pigs with different shedding levels exhibit distinct clinical, peripheral cytokine and transcriptomic immune response phenotypes. Innate Immun. (2015) 21:227–41. 10.1177/1753425914525812 24632525

[27] Osterberg J Wallgren P. Effects of a challenge dose of *Salmonella* Typhimurium or *Salmonella* Yoruba on the patterns of excretion and antibody responses of pigs. Vet Rec. (2008) 162:580–6. 10.1136/vr.162.18.580 18453377

[28] Osterberg J Lewerin SS Wallgren P. Patterns of excretion and antibody responses of pigs inoculated with *Salmonella* Derby and *Salmonella* Cubana. Vet Rec. (2009) 165:404–8. 10.1136/vr.165.14.404 19801593

[29] Vieira-Pinto M Temudo P Martins C. Occurrence of *Salmonella* in the ileum, ileocolic lymph nodes, tonsils, mandibular lymph nodes and carcasses of pigs slaughtered for consumption. J Vet Med Ser B Infect Dis Vet Public Health. (2005) 52:476–81. 10.1111/j.1439-0450.2005.00892.x 16364024

[30] Cote S Letellier A Lessard L Quessy S. Distribution of *Salmonella* in tissues following natural and experimental infection in pigs. Can J Vet Res. (2004) 68:241–8. 15581217 PMC1111353

[31] Guerra JBP Yamatogi RS Possebon FS Fernandes SA Tiba-Casas MR Lara GHB Frequency, serotyping and antimicrobial resistance pattern of *Salmonella* from feces and lymph nodes of pigs. Pesquisa Vet Brasileira. (2016) 36:1165–70. 10.1590/s0100-736x2016001200004

[32] NARMS The National Antimicrobial Resistance Monitoring System: NARMS Integrated Report. Laurel, MD: U.S. Department of Health and Human Services, FDA (2017).

[33] Slade RD Kyriazakis I Carroll SM Reynolds FH Wellock IJ Broom LJ. Effect of rearing environment and dietary zinc oxide on the response of group-housed weaned pigs to enterotoxigenic *Escherichia coli* O149 challenge. Animal. (2011) 5:1170–8. 10.1017/S1751731111000188 22440169

[34] Crayford G Coombes JL Humphrey TJ Wigley P Monophasic expression of FliC by *Salmonella* 4, 5,12:i:-DT193 does not alter its pathogenicity during infection of porcine intestinal epithelial cells. Microbiology. (2014) 160:2507–16. 10.1099/mic.0.081349-0 25118251

[35] Seixas R Machado J Bernardo F Vilela C Oliveira M. Biofilm formation by *Salmonella enterica* serovar 1,4,5,12:i:- Portuguese isolates: a phenotypic, genotypic, and socio-geographic analysis. Curr Microbiol. (2014) 68:670–7. 10.1007/s00284-014-0523-x 24463530

[36] Tassinari E Duffy G Bawn M Burgess CM McCabe EM Lawlor PG. Microevolution of antimicrobial resistance and biofilm formation of *Salmonella* Typhimurium during persistence on pig farms. Sci Rep. (2019) 9:12. 10.1038/s41598-019-45216-w 31222015 PMC6586642

[37] Barilli E Bacci C Villa ZS Merialdi G D'Incau M Brindani F. Antimicrobial resistance, biofilm synthesis and virulence genes in *Salmonella* isolated from pigs bred on intensive farms. Ital J Food Safety. (2018) 7:131–7. 10.4081/ijfs.2018.7223 30046559 PMC6036996

[38] CLSI Performance Stnadards for Antimicrobial Disk and Dilution Susceptiblity Tests for Bacteria Isolated From Animals. CLSI Supplement VET08. Wayne, PA: Clinical and Laboratory Standards Institute (2015).

[39] Wood RL Pospischil A Rose R. Distribution of persistent *Salmonella* typhimurium infection in internal organs of swine. Am J Vet Res. (1989) 50:1015–21. 2528309

[40] Wood RL Rose R. Populations of *Salmonella* typhimurium in internal organs of experimentally infected carrier swine. Am J Vet Res. (1992) 53:653–8. 1524288

[41] Song M Liu Y Soares JA Che TM Osuna O Maddox CW. Dietary clays alleviate diarrhea of weaned pigs. J Anim Sci. (2012) 90:345–60. 10.2527/jas.2010-3662 21908641

[42] Khachatryan AR Hancock DD Besser TE Call DR. Role of calf-adapted *Escherichia coli* in maintenance of antimicrobial drug resistance in dairy calves. Appl Environ Microbiol. (2004) 70:752–7. 10.1128/AEM.70.2.752-757.2004 14766551 PMC348837

[43] Inouye M Dashnow H Raven L-A Schultz MB Pope BJ Tomita T. SRST2: rapid genomic surveillance for public health and hospital microbiology labs. Genome Med. (2014) 6:90. 10.1186/s13073-014-0090-6 25422674 PMC4237778

[44] Gupta SK Padmanabhan BR Diene SM Lopez-Rojas R Kempf M Landraud L. ARG-ANNOT, a new bioinformatic tool to discover antibiotic resistance genes in bacterial genomes. Antimicrob Agents Chemother. (2014) 58:212–20. 10.1128/AAC.01310-13 24145532 PMC3910750

[45] Carattoli A Zankari E Garcia-Fernandez A Larsen MV Lund O Villa L. *In silico* detection and typing of plasmids using PlasmidFinder and plasmid multilocus sequence typing. Antimicrob Agents Chemother. (2014) 58:3895–903. 10.1128/AAC.02412-14 24777092 PMC4068535

[46] Chen L Xiong Z Sun L Yang J Jin Q. VFDB 2012 update: toward the genetic diversity and molecular evolution of bacterial virulence factors. Nucleic Acids Res. (2012) 40:D641–5. 10.1093/nar/gkr989 22067448 PMC3245122

[47] Bankevich A Nurk S Antipov D Gurevich AA Dvorkin M Kulikov AS. SPAdes: a new genome assembly algorithm and its applications to single-cell sequencing. J Comput Biol. (2012) 19:455–77. 10.1089/cmb.2012.0021 22506599 PMC3342519

[48] Lucarelli C Dionisi AM Filetici E Owczarek S Luzzi I Villa L. Nucleotide sequence of the chromosomal region conferring multidrug resistance (R-type ASSuT) in *Salmonella* Typhimurium and monophasic Salmonella Typhimurium strains. J Antimicrob Chemother. (2012) 67:111–4. 10.1093/jac/dkr391 21990047

[49] Garcia P Malorny B Rodicio MR Stephan R Hachler H Guerra B. Horizontal acquisition of a multidrug-resistance module (R-type ASSuT) is responsible for the monophasic phenotype in a widespread clone of *Salmonella* serovar 4, 5,12:i. Front Microbiol. (2016) 7:7. 10.3389/fmicb.2016.00680 27242707 PMC4861720

[50] Kim K Ehrlich A Perng V Chase JA Raybould H Li XD Algae-derived beta-glucan enhanced gut health and immune responses of weaned pigs experimentally infected with a pathogenic E-coli. Anim Feed Sci Technol. (2019) 248:114–25. 10.1016/j.anifeedsci.2018.12.004

[51] Funk JA Davies PR Nichols MA. The effect of fecal sample weight on detection of *Salmonella enterica* in swine feces. J Vet Diagn Investig. (2000) 12:412–8. 10.1177/104063870001200504 11021427

[52] Charles SD Abraham AS Trigo ET Jones GF Settje TL Reduced shedding and clinical signs of *Salmonella* Typhimurium in nursery pigs vaccinated with a *Salmonella* Choleraesuis vaccine. Swine Health Prod. (2000) 8:107–12.

[53] Boyen F Pasmans F Van Immerseel F Donne E Morgan E Ducatelle R. Porcine *in vitro* and *in vivo* models to assess the virulence of *Salmonella enterica* serovar Typhimurium for pigs. Lab Anim. (2009) 43:46–52. 10.1258/la.2007.007084 18987064

[54] Chisholm KI Ida KK Davies AL Tachtsidis I Papkovsky DB Dyson A. Hypothermia protects brain mitochondrial function from hypoxemia in a murine model of sepsis. J Cereb Blood Flow Metab. (2016) 36:1955–64. 10.1177/0271678X15606457 26661160 PMC5094296

[55] Schieber AMP Ayres JS. Thermoregulation as a disease tolerance defense strategy. Pathog Dis. (2016) 74:15. 10.1093/femspd/ftw106 27815313 PMC5975229

[56] Schwarz P Kich JD Coldebella A Seyboth L Romeiro C Corbellini LG Frequency of *Salmonella* seropositive pigs in farms affected by different severity levels of the post-weaning multisystemic wasting syndrome. Acta Sci Vet. (2010) 38:127–32. 10.22456/1679-9216.16609

[57] Leite FL Vasquez E Gebhart CJ Isaacson RE The effects of *Lawsonia intracellularis, Salmonella enterica* serovar Typhimurium and co-infection on IL-8 and TNF alpha expression in IPEC-J2 cells. Vet Microbiol. (2019) 231:76–9. 10.1016/j.vetmic.2019.02.036 30955828

[58] Kaldhone PR Han J Deck J Khajanchi B Nayak R Foley SL. Evaluation of the genetics and functionality of plasmids Incompatibility Group I1-positive *Salmonella enterica*. Foodbourne Pathog Dis. (2018) 15:168–76. 10.1089/fpd.2017.2332 29265877

[59] Elnekave E Hong S Mather AE Boxrud D Taylor AJ Lappi V. *Salmonella enterica* serotype 4,5 12:i:- in swine in the United States Midwest: an emerging multidrug-resistant clade. Clin Infect Dis. (2018) 66:877–85. 10.1093/cid/cix909 29069323

[60] Kuijpers AFA Marinovic AAB Wijnands LM Delfgou-van Asch EHM van Hoek A Franz E. Phenotypic prediction: linking *in vitro* virulence to the genomics of 59 *Salmonella enterica* strains. Front Microbiol. (2019) 9:13. 10.3389/fmicb.2018.03182 30687242 PMC6333659

[61] Gunnarsdottir R Muller K Jensen PE Jenssen PD Villumsen A. Effect of long-term freezing and freeze-thaw cycles on indigenous and inoculated microorganisms in dewatered blackwater. Environ Sci Technol. (2012) 46:12408–16. 10.1021/es3018489 23113759

[62] Garcia-Feliz C Collazos JA Carvajal A Herrera S Echeita MA Rubio P. Antimicrobial resistance of *Salmonella enterica* isolates from apparently healthy and clinically ill finishing pigs in Spain. Zoonoses Public Health. (2008) 55:195–205. 10.1111/j.1863-2378.2008.01110.x 18387141

[63] Lindsay D Dubey J Santin-Duran M Coccidia and other protozoa. In: Zimmerman J Karriker L Ramirez A Schwartz K Stevenson G Zhang J, editors. Diseases of Swine, 11 ed Hoboken, NJ: John Wiley and Sons, Inc. (2019). p. 1015–27.

