# Supplementary material for: Pathogenicity and Competitive Fitness of Salmonella enterica Serovar 4,[5],12:i:- Compared to Salmonella Typhimurium and Salmonella Derby in Swine
Source: Front Vet Sci. 2020 Jan 30;6:502. doi: 10.3389/fvets.2019.00502 (PMC7002397; doi:10.3389/fvets.2019.00502)
Supplement: Supplementary file 1 [file Data_Sheet_1.docx]

Supplementary Material

# Supplementary Figures

| **Fecal Score** | **Description** | **Image** |
| --- | --- | --- |
| **1** | Firm and dry | **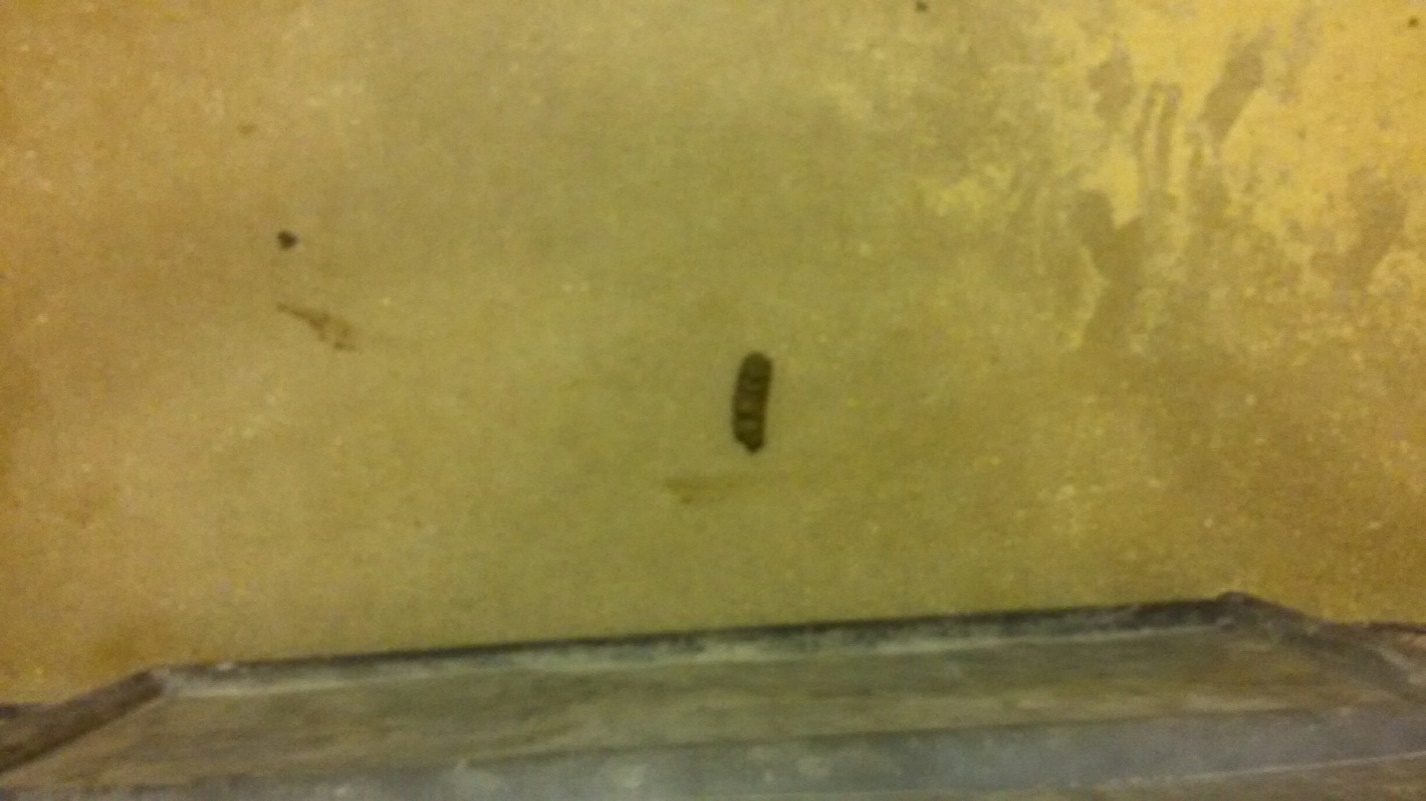** |
| **2** | Firm and moist | **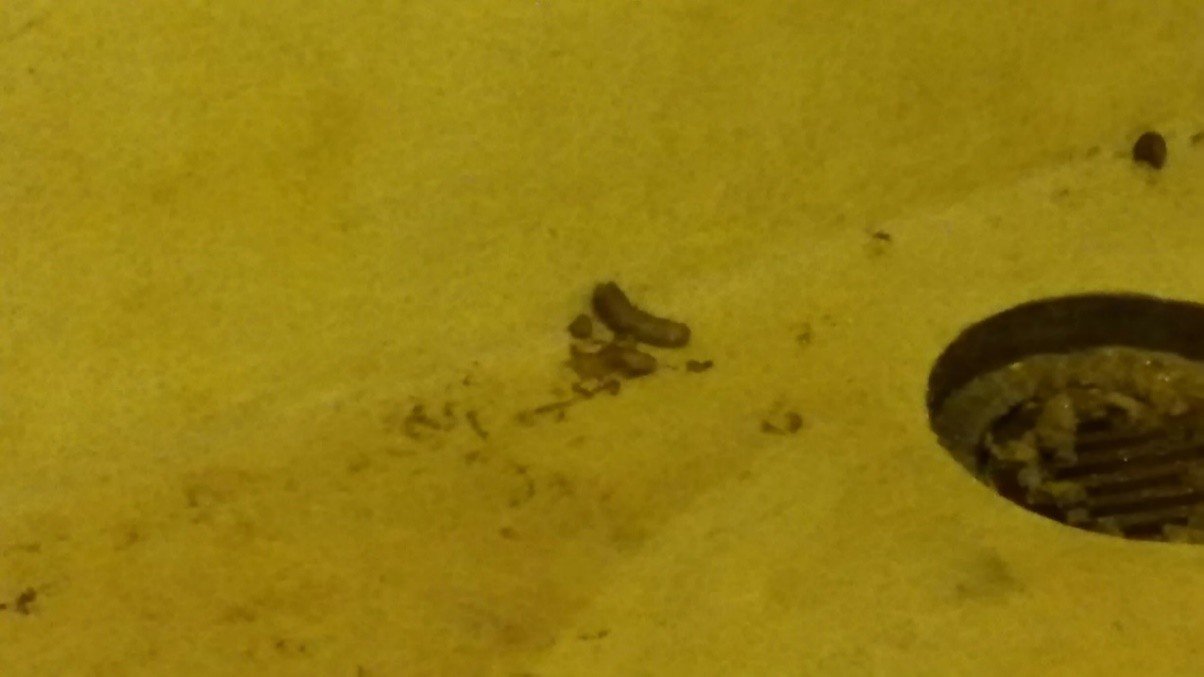** |
| **3** | “Cow-pie” appearance; increasingly moist with no clumping on digital rectal exam | **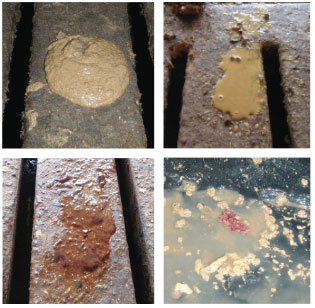** |
| **4** | “Pancake” appearance; increasingly moist, flattens out on floor | **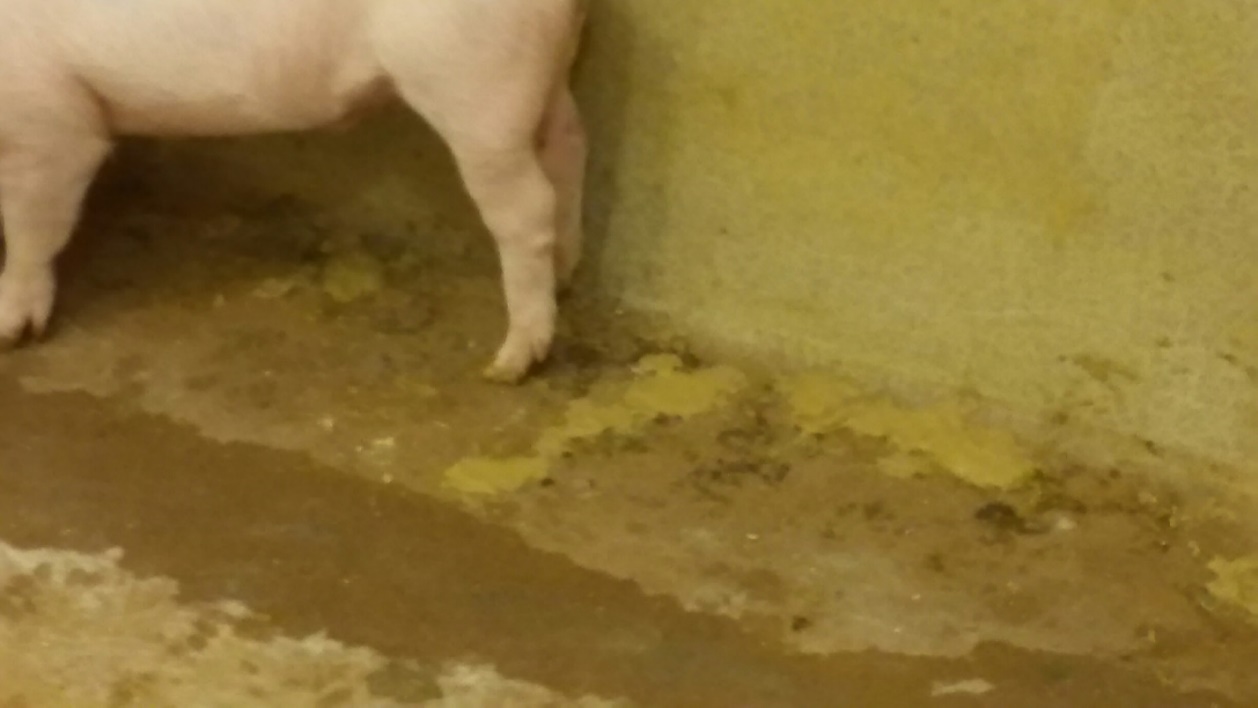** |
| **5** | Consistency resembles water | **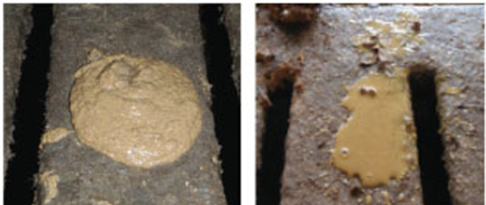** |

**Supplemental Figure 1. Depiction and explanation of fecal scoring system utilized for all three animal studies to determine the level of diarrhea.**


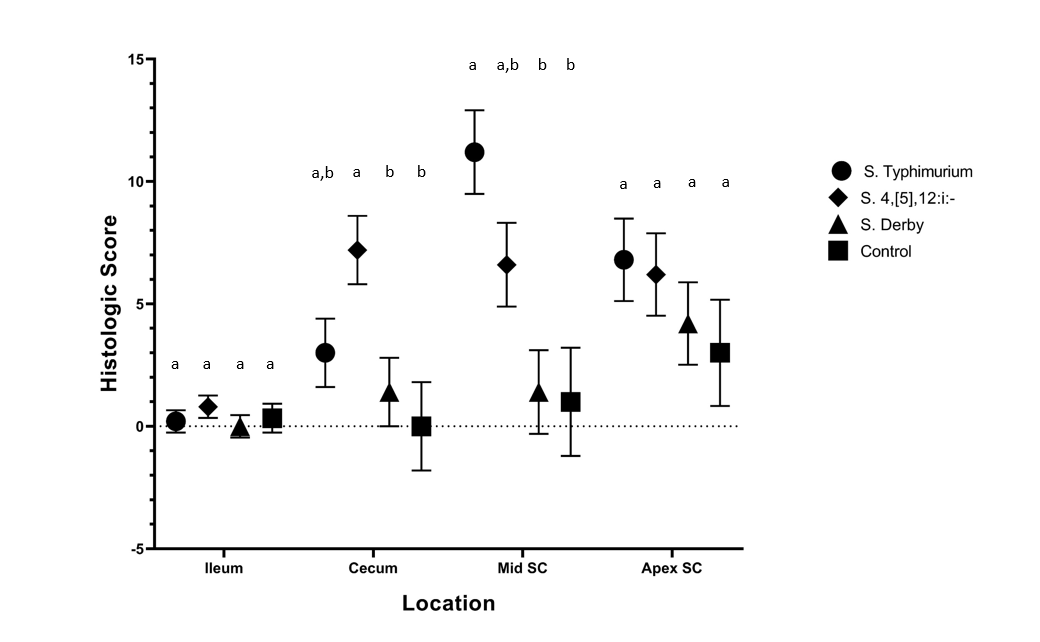


**Supplemental Figure 2A.**


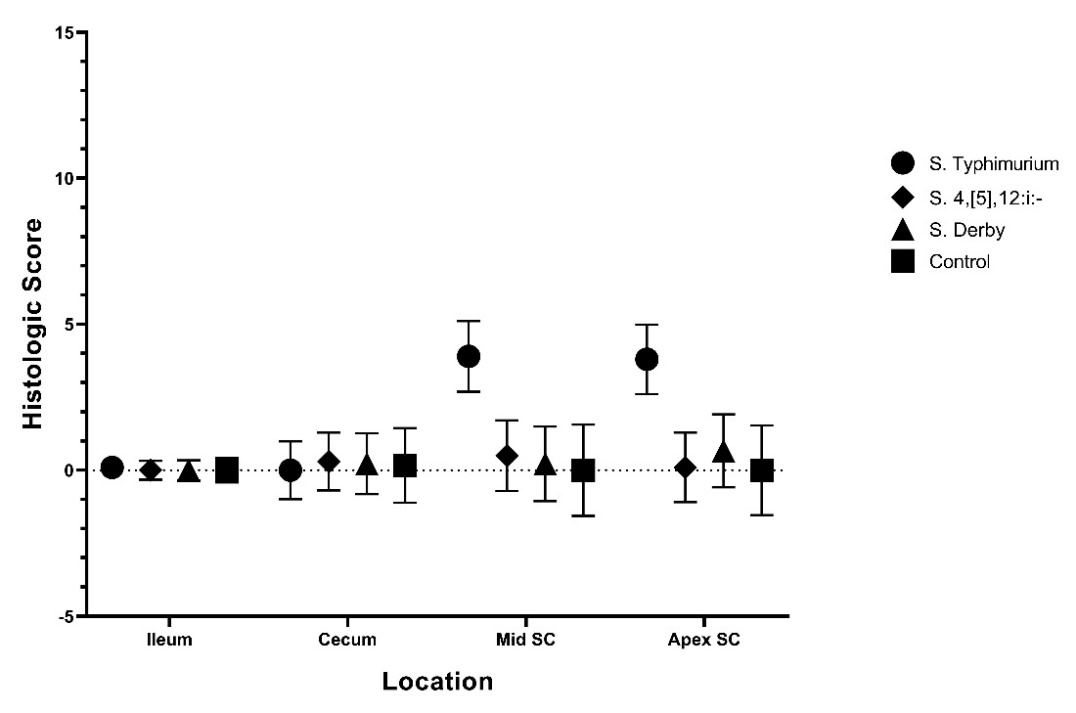


**Supplemental Figure 2B.**

**Supplemental Figure 2.** **Comparison of histologic lesion scores from pigs inoculated with *Salmonella* serovars 4,[5],12:i:-, Typhimurium, and Derby and non-inoculated control pigs in animal study #2.** Histologic lesion scores represent a summary of the ulceration, neutrophil infiltration, crypt elongation and abscessation, and submucosal inflammation at the time of necropsy on (2A) DPI 4, and (2B) DPI 28. Mean and standard error are represented by the symbols and vertical lines, respectively. Different letters indicate statistically significant differences between serovar groups, separated by tissue location (p<0.05).


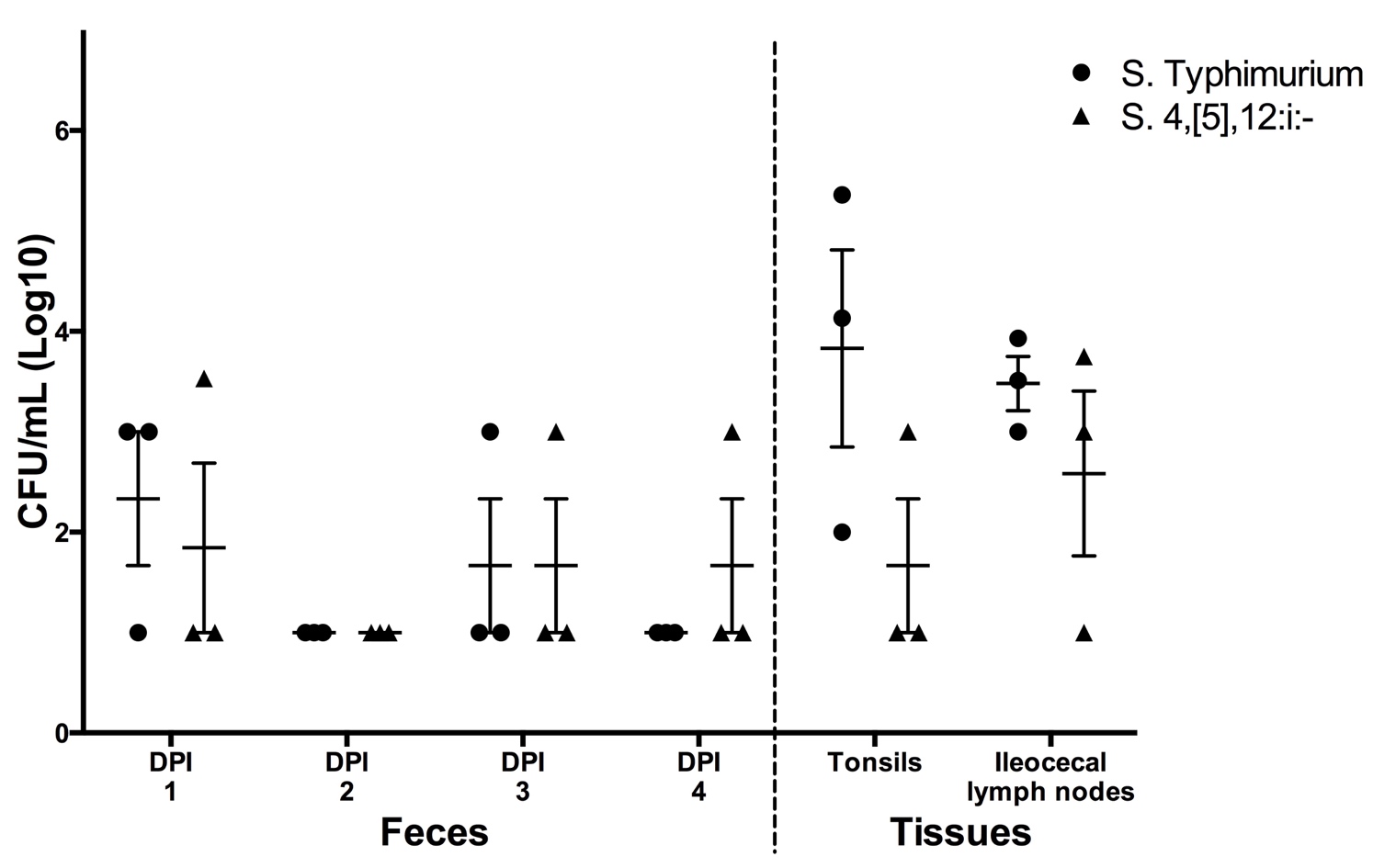


**Supplemental Figure 3. Comparison of quantitative culture results for *Salmonella* in feces, ileocecal lymph nodes and tonsils collected from singly-inoculated pigs in animal study #3.** The mean log_10_ CFU/mL and standard error are represented by the horizontal and vertical lines, respectively.

# Supplemental Tables

**Supplemental Table 1.** Comparison of *Salmonella* culture results in feces collected from pigs inoculated with *Salmonella* serovars 4,[5],12:i:-, Typhimurium and Derby in animal study #2.

|  | **Fecal culture positives*** | | | | | |
| --- | --- | --- | --- | --- | --- | --- |
| **Inoculum** | **DPI** | | | | | |
|  | **2** | **4** | **7** | **14** | **21** | **28** |
| **S. 4,[5],12:i:-** | 100% | 73% | 90% | 40% | 30% | 0% |
|  | 20/20 | 11/15 | 9/10 | 4/10 | 3/10 | 0/10 |
| **S. Typhimurium** | 95% | 93% | 60% | 40% | 20% | 20% |
|  | 19/20 | 14/15 | 6/10 | 4/10 | 2/10 | 2/10 |
| **S. Derby** | 95% | 79% | 67% | 11% | 11% | 0% |
|  | 18/19 | 11/14 | 6/9 | 1/9 | 1/9 | 0/9 |

DPI = days post inoculation

*Positives were positive for *Salmonella* from quantitative and/or enrichment culture

**Supplemental Table 2.** Results of MLST typing based on whole genome sequencing of isolates of *Salmonella* utilized for inoculation of pigs in animal studies #2 and #3.

|  |  |  | **Loci** | | | | | | |
| --- | --- | --- | --- | --- | --- | --- | --- | --- | --- |
| **Isolate** | **Serovar** | **ST** | **aroC** | **dnaN** | **hemD** | **hisD** | **purE** | **sucA** | **thrA** |
| ISU-SAL240-15 | 4,5,12:i:- | 34 | 10 | 19 | 12 | 9 | 5 | 9 | 2 |
| ISU-SAL245-16 | 4,5,12:i:- | 34 | 10 | 19 | 12 | 9 | 5 | 9 | 2 |
| ISU-SAL243-14 | Typhimurium | 19 | 10 | 7 | 12 | 9 | 5 | 9 | 2 |
| ISU-SAL244-16 | Typhimurium | 19 | 10 | 7 | 12 | 9 | 5 | 9 | 2 |
| ISU-SAL242-16 | Derby | 40 | 19 | 20 | 3 | 20 | 5 | 22 | 22 |

ST = sequence type
